# Supplementary material for: Discovery and application of insertion-deletion (INDEL) polymorphisms for QTL mapping of early life-history traits in Atlantic salmon
Source: BMC Genomics. 2010 Mar 8;11:156. doi: 10.1186/1471-2164-11-156 (PMC2838853; doi:10.1186/1471-2164-11-156)
Supplement: Additional file 2 — Information on developed 76 locus single-run INDEL panel in Atlantic salmon. Information on fluorescence labeling, primer concentrations, PCR pooling and links to alignments, INDEL motifs and GENESCAN (Burge and Karlin 1997) predictions of genes/exons are available in html format. [file 1471-2164-11-156-S2.ZIP › Additionalfile2/table905.html]

Contig Alignment

```
Base	A B C D E F G H I J K L M N O P Q R S T U V W X Y Z a b c d e f g h i j k l m n o p q r s t u v w x y z A B C D E F G H I J K L M N O P Q R S T U V W   
1	. A . . . . . . . . . . . . . . . . . A . . . . . A . . . . . . A . . . . . . . . . . . . . . . . . . . . . . . . . . . . . . . . . . . . . . . . . .    
2	. T . . . . . . . . . . . . . . . . . T . . . . . T . . . . . . T . . . . . . . . . . . . . . . . . . . . . . . . . . . . . . . . . . . . . . . . . .    
3	. G . . . . . . . . . . . . . . . . . G . . . . . G . . . . . . G G . . . . . . . . . . . . . . . . . . . . . . . . . . . . . . . . . . . . . . . . .    
4	. G . . . . . . . . . . . . . . . . . G . . . . . G . . . . . . G G . . . . . . . . . . . . . . . . . . . . . . . . . . . . . . . . . . . . . . . . .    
5	. C . . . . . . . . . . . . . . . . . C . . . . . C . . . . . . C C . . . . . . . . . . . . . . . . . . . . . . . . . . . . . . . . . . . . . . . . .    
6	. C . . . . . . . . . . . . . . . . . C . . . . . C . . . . . . C C . . . . . . . . . . . . . . . . . . . . . . . . . . . . . . . . . . . . . . . . .    
7	. A . . . . . . . . . . . . . . . . . A . . . . . A . . . . . . A A . . . . . . . . . . . . . . . . . . . . . . . . . . . . . . . . . . . . . . . . .    
8	. G . . . . . . . . . . . . . . . . . G . . . . . G . . . . . . G G . . . . . . . . . . . . . . . . . . . . . . . . . . . . . . . . . . . . . . . . .    
9	. C . . . . . . . . . . . . . . . . . C . . . . . C . . . . . . C C . . . . . . . . . . . . . . . . . . . . . . . . . . . . . . . . . . . . . . . . .    
10	. A . . . . . . . . . . . . . . . . . A . . . . . A . . . . . . A A . . . . . . . . . . . . . . . . . . . . . . . . . . . . . . . . . . . . . . . . .    
11	. G . . . . . . . . . . . . . . . . . G . . . . . G . . . . . . G G . . . . . . . . . . . . . . . . . . . . . . . . . . . . . . . . . . . . . . . . .    
12	. C . . . . . . . . . . . . . . . . . C . . . . . C . . . . . . C C . . . . . . . . . . . . . . . . . . . . . . . . . . . . . . . . . . . . . . . . .    
13	. T . . . . . . . . . . . . . . . . . T . . . . . T T . . . . . T T . . . . . . . . . . . . . . . . . . . . . . . . . . . . . . . . . . . . . . . . .    
14	. T . . . . . . . . . . . . . . . . . T . . . . . T T . . . . . T T . . . . . . . . . . . . . . . . . . . . . . . . . . . . . . . . . . . . . . . . .    
15	. T . . . . . . . . . . . . . . . . . T . . . . . T T . . . . . T T . . . . . . . . . . . . . . . . . . . . . . . . . . . . . . . . . . . . . . . . .    
16	. T . . . . . . . . . . . . . . . . . T . . . . . T T . . . . . T T . . . . . . . . . . . . . . . . . . . . . . . . . . . . . . . . . . . . . . . . .    
17	. A A . A . . A A A . . A A . . . A . A . . A A A A A . A . A A A A A A A A . . . . . . . . . . . . . . . . . . . . . . . . . . . . . . . . . . . . .    
18	. T T . T . . T T T . . T T . . . T . T . . T T T T T . T . T T T T T T T T . . . . . . . . . . . . . . . . . . . . . . . . . . . . . . . . . . . . .    
19	. A A . A . . A A A . . A A . . . A . A . . A A A A A . A . A A A A A A A A . . . . . . . . . . . . . . . . . . . . . . . . . . . . . . . . . . . . .    
20	. A A . A . . A A A . . A A . . . A . A . . A A A A A A A . A A A A A A A A . . . . . . . . . . . . . . . . . . . . . . . . . . . . . . . . . . . . .    
21	. A A . A . . A A A . . A A . . . A . A . . A A A A A A A . A A A A A A A A . . . . . . . . . . . . . . . . . . . . . . . . . . . . . . . . . . . . .    
22	. A A . A . . A A A . . A A . . . A . A . . A A A A A A A . A A A A A A A A . . . . . . . . . . . . . . . . . . . . . . . . . . . . . . . . . . . . .    
23	. G G . G . . G G G . . G G . . . G . G . . G G G G G G G . G G G G G G G G . . . . . . . . . . . . . . . . . . . . . . . . . . . . . . . . . . . . .    
24	. G G . G . . G G G . . G G . . . G . G . . G G G G G G G . G G G G G G G G . . . . . . . . . . . . . . . . . . . . . . . . . . . . . . . . . . . . .    
25	. A A . A . . A A A . . A A . . . A . A . . A A A A A A A . A A A A A A A A . . . . . . . . . . . . . . . . . . . . . . . . . . . . . . . . . . . . .    
26	. C C . C . . C C C . . C C . . . C . C . . C C C C C C C . C C C C C C C C . . . . . . . . . . . . . . . . . . . . . . . . . . . . . . . . . . . . .    
27	. C C . C . . C C C . . C C . . . C . C . . C C C C C C C . C C C C C C C C . . . . . . . . . . . . . . . . . . . . . . . . . . . . . . . . . . . . .    
28	. A A . A . . A A A . . A A . . . A . A . . A A A A A A A . A A A A A A A A . . . . . . . . . . . . . . . . . . . . . . . . . . . . . . . . . . . . .    
29	. T T . T . . T T T . . T T . . . T . T . . T T T T T T T . T T T T T T T T . . . . . . . . . . . . . . . . . . . . . . . . . . . . . . . . . . . . .    
30	. T T . T . . T T T . . T T . . . T . T . . T T T T T T T . T T T T T T T T . . . . . . . . . . . . . . . . . . . . . . . . . . . . . . . . . . . . .    
31	. T T . T . . T T T . . T T . . . T . T . . T T T T T T T . T T T T T T T T . . . . . . . . . . . . . . . . . . . . . . . . . . . . . . . . . . . . .    
32	. A A . A . . A A A . . A A . . . A . A . . A A A A A A A . A A A A A A A A . . . . . . . . . . . . . . . . . . . . . . . . . . . . . . . . . . . . .    
33	. T T . T . . T T T . . T T . . . T . T . . T T T T T T T . T T T T T T T T . . . . . . . . . . . . . . . . . . . . . . . . . . . . . . . . . . . . .    
34	. T T . T . . T T T . . T T . . . T . T . . T T T T T T T . T T T T T T T T . . . . . . . . . . . . . . . . . . . . . . . . . . . . . . . . . . . . .    
35	. T T . T . . T T T . . T T . . . T . T . . T T T T T T T . T T T T T T T T . . . . . . . . . . . . . . . . . . . . . . . . . . . . . . . . . . . . .    
36	. A A . A A . A A A . A A A . . . A . A A . A A A A A A A . A A A A A A A A . . . . . . . . . . . . . . . . . . . . . . . . . . . . . . . . . . . . .    
37	. A A . A A . A A A . A A A . . . A . A A . A A A A A A A . A A A A A A A A . . . . . . . . . . . . . . . . . . . . . . . . . . . . . . . . . . . . .    
38	. C C . C C . C C C . C C C . . . C C C C . C C C C C C C . C C C C C C C C . . . . . . . . . . . . . . . . . . . . . . . . . . . . . . . . . . . . .    
39	. A A . A A . A A A . A A A . . . A A A A . A A A A A A A . A A A A A A A A . . . . . . . . . . . . . . . . . . . . . . . . . . . . . . . . . . . . .    
40	. G G . G G . G G G . G G G . . . G G G G . G G G G G G G . G G G G G G G G . . . . . . . . . . . . . . . . . . . . . . . . . . . . . . . . . . . . .    
41	. A A . A A . A A A . A A A . . A A A A A . A A A A A A A . A A A A A A A A . . . . . . . . . . . . . . . . . . . . . . . . . . . . . . . . . . . . .    
42	. A A . A A . A A A . A A A . . A A A A A . A A A A A A A . A A A A A A A A . . . . . . . . . . . . . . . . . . . . . . . . . . . . . . . . . . . . .    
43	. A A . A A . A A A . A A A . . A A A A A . A A A A A A A . A A A A A A A A . . . . . . . . . . . . . . . . . . . . . . . . . . . . . . . . . . . . .    
44	. A A . A A . A A A . A A A . . A A A A A . A A A A A A A . A A A A A A A A . . . . . . . . . . . . . . . . . . . . . . . . . . . . . . . . . . . . .    
45	. T T . T T . T T T . T T T . . T T T T T . T T T T T T T . T T T T T T T T . . . . . . . . . . . . . . . . . . . . . . . . . . . . . . . . . . . . .    
46	. A A . A A . A A A . A A A . . A A A A A . A A A A A A A . A A A A A A A A . . . . . . . . . . . . . . . . . . . . . . . . . . . . . . . . . . . . .    
47	. G G . G G . G G G . G G G . . G G G G G . G G G G G G G . G G G G G G G G . . . . . . . . . . . . . . . . . . . . . . . . . . . . . . . . . . . . .    
48	C C C . C C . C C C . C C C . . C C C C C . C C C C C C C . C C C C C C C C . . . . . . . . . . . . . . . . . . . . . . . . . . . . . . . . . . . . .    
49	A A A . A A . A A A . A A A . . A A A A A . A A A A A A A . A A A A A A A A . . . . . . . . . . . . . . . . . . . . . . . . . . . . . . . . . . . . .    
50	A A A . A A . A A A . A A A . . A A A A A . A A A A A A A . A A A A A A A A . . . . . . . . . . . . . . . . . . . . . . . . . . . . . . . . . . . . .    
51	T T T . T T . T T T . T T T . . T T T T T . T T T T T T T . T T T T T T T T . . . . . . . . . . . . . . . . . . . . . . . . . . . . . . . . . . . . .    
52	T T T . T T . T T T . T T T . . T T T T T . T T T T T T T . T T T T T T T T . . . . . . . . . . . . . . . . . . . . . . . . . . . . . . . . . . . . .    
53	T T T . T T . T T T . T T T . . T T T T T . T T T T T T T . T T T T T T T T . . . . . . . . . . . . . . . . . . . . . . . . . . . . . . . . . . . . .    
54	A A A . A A . A A A . A A A . . A A A A A . A A A A A A A . A A A A A A A A . . . . . . . . . . . . . . . . . . . . . . . . . . . . . . . . . . . . .    
55	T T T . T T . T T T . T T T . . T T T T T . T T T T T T T . T T T T T T T T . . . . . . . . . . . . . . . . . . . . . . . . . . . . . . . . . . . . .    
56	T T T . T T . T T T . T T T . . T T T T T . T T T T T T T . T T T T T T T T . . . . . . . . . . . . . . . . . . . . . . . . . . . . . . . . . . . . .    
57	T T T . T T . T T T . T T T . . T T T T T . T T T T T T T . T T T T T T T T . . . . . . . . . . . . . . . . . . . . . . . . . . . . . . . . . . . . .    
58	G G G . G G . G G G . G G G . . G G G G G . G G G G G G G . G G G G G G G G . . . . . . . . . . . . . . . . . . . . . . . . . . . . . . . . . . . . .    
59	A A A . A A . A A A . A A A . . A A A A A . A A A A A A A . A A A A A A A A . . . . . . . . . . . . . . . . . . . . . . . . . . . . . . . . . . . . .    
60	C C C . C C . C C C . C C C . . C C C C C . C C C C C C C . C C C C C C C C . . . . . . . . . . . . . . . . . . . . . . . . . . . . . . . . . . . . .    
61	A A A . A A . A A A . A A A . . A A A A A . A A A A A A A . A A A A A A A A . . . . . . . . . . . . . . . . . . . . . . . . . . . . . . . . . . . . .    
62	G G G . G G . G G G . G G G . . G G G G G . G G G G G G G . G G G G G G G G . . . . . . . . . . . . . . . . . . . . . . . . . . . . . . . . . . . . .    
63	A A A . A A . A A A . A A A . . A A A A A . A A A A A A A . A A A A A A A A . . . . . . . . . . . . . . . . . . . . . . . . . . . . . . . . . . . . .    
64	A A A . A A . A A A . A A A . . A A A A A . A A A A A A A . A A A A A A A A . . . . . . . . . . . . . . . . . . . . . . . . . . . . . . . . . . . . .    
65	T T T . T T . T T T . T T T . . T T T T T . T T T T T T T . T T T T T T T T . . . . . . . . . . . . . . . . . . . . . . . . . . . . . . . . . . . . .    
66	C C C . C C . C C C . C C C . . C C C C C . C C C C C C C . C C C C C C C C C . . . . . . . . . . . . . . . . . . . . . . . . . . . . . . . . . . . .    
67	T T T . T T . T T T . T T T . . T T T T T . T T T T T T T . T T T T T T T T T . . . . . . . . . . . . . . . . . . . . . . . . . . . . . . . . . . . .    
68	A A A . A A . A A A . A A A . . A A A A A . A A A A A A A . A A A A A A A A A . . . . . . . . . . . . . . . . . . . . . . . . . . . . . . . . . . . .    
69	A A A . A A . A A A . A A A . . A A A A A . A A A A A A A . A A A A A A A A A . . . . . . . . . . . . . . . . . . . . . . . . . . . . . . . . . . . .    
70	A A A . A A . A A A . A A A . . A A A A A . A A A A A A A . A A A A A A A A A . . . . . . . . . . . . . . . . . . . . . . . . . . . . . . . . . . . .    
71	A A A . A A . A A A . A A A . . A A A A A . A A A A A A A . A A A A A A A A A . . . . . . . . . . . . . . . . . . . . . . . . . . . . . . . . . . . .    
72	C C C . C C . C C C . C C C . . C C C C C . C C C C C C C . C C C C C C C C C . . . . . . . . . . . . . . . . . . . . . . . . . . . . . . . . . . . .    
73	C C C . C C . C C C . C C C . . C C C C C . C C C C C C C . C C C C C C C C C . . . . . . . . . . . . . . . . . . . . . . . . . . . . . . . . . . . .    
74	T T T T T T . T T T . T T T . . T T T T T . T T T T T T T . T T T T T T T T T . . . . . . . . . . . . . . . . . . . . . . . . . . . . . . . . . . . .    
75	C C C C C C . C C C . C C C . . C C C C C . C C C C C C C . C C C C C C C C C . . . . . . . . . . . . . . . . . . . . . . . . . . . . . . . . . . . .    
76	A A A A A A . A A A . A A A . . A A A A A . A A A A A A A . A A A A A A A A A . . . . . . . . . . . . . . . . . . . . . . . . . . . . . . . . . . . .    
77	A A A A A A . A A A . A A A . . A A A A A . A A A A A A A . A A A A A A A A A . . . . . . . . . . . . . . . . . . . . . . . . . . . . . . . . . . . .    
78	G G G G G G . G G G . G G G . . G G G G G . G G G G G G G . G G G G G G G G G . . . . . . . . . . . . . . . . . . . . . . . . . . . . . . . . . . . .    
79	G T T T G T . T G T . T T T . . G T T T T . T T T T T T T . T T T T T T T T T . . . . . . . . . . . . . . . . . . . . . . . . . . . . . . . . . . . .    
80	G G G G G G . G G G . G G G . . G G G G G . G G G G G G G . G G G G G G G G G . . . . . . . . . . . . . . . . . . . . . . . . . . . . . . . . . . . .    
81	A A A A A A . A A A . A A A . . A A A A A . A A A A A A A . A A A A A A A A A . . . . . . . . . . . . . . . . . . . . . . . . . . . . . . . . . . . .    
82	A A A A A A . A A A . A A A . . A A A A A . A A A A A A A . A A A A A A A A A . . . . . . . . . . . . . . . . . . . . . . . . . . . . . . . . . . . .    
83	T T T T T T . T T T . T T T . . T T T T T . T T T T T T T . T T T T T T T T T . . . . . . . . . . . . . . . . . . . . . . . . . . . . . . . . . . . .    
84	T T T T T T . T T T . T T T . . T T T T T . T T T T T T T . T T T T T T T T T . . . . . . . . . . . . . . . . . . . . . . . . . . . . . . . . . . . .    
85	T T T T T T . T T T . T T T . . T T T T T . T T T T T T T . T T T T T T T T T . . . . . . . . . . . . . . . . . . . . . . . . . . . . . . . . . . . .    
86	G G G G G G . G G G . G G G G . G G G G G . G G G G G G G . G G G G G G G G G . . . . . . . . . . . . . . . . . . . . . . . . . . . . . . . . . . . .    
87	T T T T T T . T T T T T T T T . T T T T T . T T T T T T T . T T T T T T T T T . . . . . . . . . . . . . . . . . . . . . . . . . . . . . . . . . . . .    
88	A A A A A A . A A A A A A A A . A A A A A . A A A A A A A . A A A A A A A A A . . . . . . . . . . . . . . . . . . . . . . . . . . . . . . . . . . . .    
89	T T T T T T . T T T T T T T T . T T T T T . T T T T T T T . T T T T T T T T T . . . . . . . . . . . . . . . . . . . . . . . . . . . . . . . . . . . .    
90	G G G G G G . G G G G G G G G . G G G G G . G G G G G G G . G G G G G G G G G . . . . . . . . . . . . . . . . . . . . . . . . . . . . . . . . . . . .    
91	A A A A A A . A A A A A A A A . A A A A A . A A A A A A A . A A A A A A A A A . . . . . . . . . . . . . . . . . . . . . . . . . . . . . . . . . . . .    
92	A A A A A A . A A A A A A A A . A A A A A . A A A A A A A . A A A A A A A A A . . . . . . . . . . . . . . . . . . . . . . . . . . . . . . . . . . . .    
93	T T T T T T . T T T T T T T T . T T T T T . T T T T T T T . T T T T T T T T T . . . . . . . . . . . . . . . . . . . . . . . . . . . . . . . . . . . .    
94	T T T T T T . T T T T T T T T . T T T T T . T T T T T T T . T T T T T T T T T . . . . . . . . . . . . . . . . . . . . . . . . . . . . . . . . . . . .    
95	A A A A A A . A A A A A A A A . A A A A A . A A A A A A A . A A A A A A A A A . . . . . . . . . . . . . . . . . . . . . . . . . . . . . . . . . . . .    
96	C C C C C C . C C C C C C C C . C C C C C . C C C C C C C . C C C C C C C C C . . . . . . . . . . . . . . . . . . . . . . . . . . . . . . . . . . . .    
97	C C C C C C . C C C C C C C C . C C C C C . C C C C C C C . C C C C C C C C C . . . . . . . . . . . . . . . . . . . . . . . . . . . . . . . . . . . .    
98	A A A A A A . A A A A A A A A . A A A A A . A A A A A A A . A A A A A A A A A . . . . . . . . . . . . . . . . . . . . . . . . . . . . . . . . . . . .    
99	T T T T T T . T T T T T T T T . T T T T T . T T T T T T T . T T T T T T T T T . . . . . . . . . . . . . . . . . . . . . . . . . . . . . . . . . . . .    
100	T T T T T T . T T T T T T T T . T T T T T . T T T T T T T . T T T T T T T T T . . . . . . . . . . . . . . . . . . . . . . . . . . . . . . . . . . . .    
101	T T T T T T . T T T T T T T T . T T T T T . T T T T T T T . T T T T T T T T T . . . . . . . . . . . . . . . . . . . . . . . . . . . . . . . . . . . .    
102	T T T T T T . T T T T T T T T . T T T T T . T T T T T T T . T T T T T T T T T . . . T . . . . . . . . . . . . . . . . . . . . . . . . . . . . . . . .    
103	T T T T T T . T T T T T T T T . T T T T T . T T T T T T T . T T T T T T T T T . . . T . . . . . . . . . . . . . . . . . . . . . . . . . . . . . . . .    
104	T T T T T T . T T T T T T T T . T T T T T . T T T T T T T . T T T T T T T T T . . . T . . . . . . . . . . . . . . . . . . . . . . . . . . . . . . . .    
105	A A A A A A . A A A A A A A A . A A A A A . A A A A A A A . A A A A A A A A A . . . A . . . . . . . . . . . . . . . . . . . . . . . . . . . . . . . .    
106	T T T T T T . T T T T T T T T . T T T T T . T T T T T T T . T T T T T T T T T . . . T . . . . . . . . . . . . . . . . . . . . . . . . . . . . . . . .    
107	G G G G G G . G G G G G G G G . G G G G G . G G G G G G G . G G G G G G G G G . . . G . . . . . . . . . . . . . . . . . . . . . . . . . . . . . . . .    
108	A A A A A A . A A A A A A A A . A A A A A . A A A A A A A . A A A A A A A A A . . . A . . . . . . . . . . . . . . . . . . . . . . . . . . . . . . . .    
109	A A A A A A . A A A A A A A A . A A A A A . A A A A A A A . A A A A A A A A A . . . A . . . . . . . . . . . . . . . . . . . . . . . . . . . . . . . .    
110	T T T T T T . T T T T T T T T . T T T T T . T T T T T T T . T T T T T T T T T . . . T . . . . . . . . . . . . . . . . . . . . . . . . . . . . . . . .    
111	A A A A A A . A A A A A A A A . A A A A A . A A A A A A A . A A A A A A A A A . . . A . . . . . . . . . . . . . . . . . . . . . . . . . . . . . . . .    
112	C C C C C C . C C C C C C C C . C C C C C . C C C C C C C . C C C C C C C C C . . . C . . . . . . . . . . . . . . . . . . . . . . . . . . . . . . . .    
113	G G G G G G . G G G G G G G G . G G G G G . G G G G G G G . G G G G G G G G G . . . G . . . . . . . . . . . . . . . . . . . . . . . . . . . . . . . .    
114	T T T T T T . T T T T T T T T . T T T T T . T T T T T T T . T T T T T T T T T . . . T . . . . . . . . . . . . . . . . . . . . . . . . . . . . . . . .    
115	T T T T T T . T T T T T T T T . T T T T T . T T T T T T T . T T T T T T T T T . . . T . . . . . . . . . . . T . . . . . . . . . . . . . . . . . . . .    
116	T T T T T T . T T T T T T T T . T T T T T . T T T T T T T . T T T T T T T T T . . . T . . . . . . . . . . . T . . . . . . . . . . . . . . . . . . . .    
117	A A A A A A . A A A A A A A A . A A A A A . A A A A A A A . A A A A A A A A A . . . A . . . . . . . . . . . A . . . . . . . . . . . . . . . . . . . .    
118	G G G G G G . G G G G G G G G . G G G G G . G G G G G G G . G G G G G G G G G . . . G . . . . . . . . . . . G . . . . . . . . . . . . . . . . . . . .    
119	A A A A A A . A A A A A A A A . A A A A A . A A A A A A A . A A A A A A A A A . . . A . . . . . . . . . . . A . . . . . . . . . . . . . . . . . . . .    
120	T T T T T T . T T T T T T T T . T T T T T . T T T T T T T . T T T T T T T T T . . . T . . . . . . . . . . . T . . . . . . . . . . . . . . . . . . . .    
121	C C C C C C . C C C C C C C C . C C C C C . C C C C C C C . C C C C C C C C C . . . C . . . . . . . . . . . C . . . . . . . . . . . . . . . . . . . .    
122	A A A A A A . A A A A A A A A . A A A A A . A A A A A A A . A A A A A A A A A . . . A . . . . . . . . . . . A . . . . . . . . . . . . . . . . . . . .    
123	A A A A A A . A A A A A A A A . A A A A A . A A A A A A A . A A A A A A A A A . . . A . . . . . . . . . . . A . . . . . . . . . . . . . . . . . . . .    
124	A A A A A A . A A A A A A A A . A A A A A . A A A A A A A . A A A A A A A A A . . . A . . . . . . . . . . . A . . . . . . . . . . . . . . . . . . . .    
125	C C C C C C C C C C C C C C C . C C C C C . C C C C C C C . C C C C C C C C C . . . C . . . . . . . . . . . C . . . . . . . . . . . . . . . . . . . .    
126	T T T T T T T T T T T T T T T . T T T T T . T T T T T T T . T T T T T T T T T . . . T . . . . . . . . . . . T . . . . . . . . . . . . . . . . . . . .    
127	G G G G G G G G G G G G G G G . G G G G G . G G G G G G G . G G G G G G G G G . . . G . . . . . . . . . . . G . . . . . . . . . . . . . . . . . . . .    
128	C C C C C C C C C C C C C C C . C C C C C . C C C C C C C . C C C C C C C C C . . . C . . . . . . . . . . . C . . . . . . . . . . . . . . . . . . . .    
129	A A A A A A A A A A A A A A A . A A A A A . A A A A A A A . A A A A A A A A A . . . A . . . . . . . . . . . A . . . . . . . . . . . . . . . . . . . .    
130	G G G G G G G G G G G G G G G . A G G G G . G G G G G G G . G G G G G G G G G . . . G . . . . . G . . . . . G . . . . . . . . . . . . . . . . . . . .    
131	A A A A A A A A A A A A A A A . A A A A A . A A A A A A A . A A A A A A A A A . . . A . . . . . A . . . . . A . . . . . . . . . . . . . . . . . . . .    
132	A A A A A A A A A A A A A A A . A A A A A . A A A A A A A . A A A A A A A A A . . . A . . . . . A . . . . . A . . . . . . . . . . . . . . . . . . . .    
133	C C C C C C C C C C C C C C C . C C C C C . C C C C C C C . C C C C C C C C C . . . C . . . . . C . . . . . C . . . . . . . . . . . . . . . . . . . .    
134	A A A A A A A A A A A A A A A . A A A A A . A A A A A A A . A A A A A A A A A . . . A . . . . . A . . . . . A . . . . . . . . . . . . . . . . . . . .    
135	T T T T T T T T T T T T T T T . T T T T T . T T T T T T T . T T T T T T T T T . . . T . . . . . T . . . . . T . . . T . . . . . . . . . . . . . . . .    
136	T T T T T T T T T T T T T T T . T T T T T . T T T T T T T . T T T T T T T T T . . . T . . . . . T . . . . . T . . . T . . . . . . . . . . . . . . . .    
137	T T T T T T T T T T T T T T T . T T T T T . T T T T T T T . T T T T T T T T T . . . T . . . . . T . . . . . T . . . T . . . . . . . . . . . . . . . .    
138	T T T T T T T T T T T T T T T . T T T T T . T T T T T T T . T T T T T T T T T . . . T . . . . . T . . . . . T . . . T . . . . . . . . . . . . . . . .    
139	T T T T T T T T T T T T T T T . T T T T T . T T T T T T T . T T T T T T T T T . . . T . . . . . T . . . . . T . . . T . . . . . . . . . . . . . . . .    
140	T T T T T T T T T T T T T T T . T T T T T . T T T T T T T . T T T T T T T T T . . . T . . . . . T . . . . . T . . . T . . . . . . . . . . . . . . . .    
141	C C C C C C C C C C C C C C C . C C C C C . C C C C C C C . C C C C C C C C C . . . C . . . . . C . . . . . C . . . C . . . . . . . . . . . . . . . .    
142	C C C C C C C C C C C C C C C . C C C C C . C C C C C C C . C C C C C C C C C . . . C . . . . . C . . . . . C . . . C . . . . . . . . . . . . . . . .    
143	A A A A A A A A A A A A A A A . A A A A A . A A A A A A A . A A A A A A A A A . . . A . . . . . A . . . . . A . . . A . . . . . . . . . . . . . . . .    
144	C C C C C C C C C C C C C C C . C C C C C . C C C C C C C . C C C C C C C C C . . . C . . . . . C . . . . . C . . . C . . . . . . . . . . . . . . . .    
145	A A A A A A A A A A A A A A A . A A A A A . A A A A A A A . A A A A A A A A A . . . A . . . . . A . . . . . A . . . A A . . . . . . . . . . . . . . .    
146	G G G G G G G G G G G G G G G . G G G G G . G G G G G G G . G G G G G G G G G . . . G . . . . . G . . . . . G . . . G G . . . . . . . . . . . . . . .    
147	A A A A A A A A A A A A A A A . A A A A A . A A A A A A A . A A A A A A A A A . . . A . . . . . A . . . . . A . . . A A . . . . . . . . . . . . . . .    
148	G G G G G G G G G G G G G G G . G G G G G . G G G G G G G . G G G G G G G G G . . . G . . . . . G . . . . . G . . . G G . . . . . . . . . . . . . . .    
149	A A A A A A A A A A A A A A A . A A A A A . A A A A A A A . A A A A A A A A A . . . A . . . . . A . . . . . A . . . A A . . . . . . . . . . . . . . .    
150	T T T T T T T T T T T T T T T . T T T T T . T T T T T T T . T T T T T T T T T . . . T . . . . . T . . . . . T . . . T T . . . . . . . . . . . . . . .    
151	T T T T T T T T T T T T T T T . T T T T T . T T T T T T T . T T T T T T T T T . . . T . . . . . T . . . . . T . . . T T . . . . . . . . . . . . . . .    
152	G G G G G G G G G G G G G G G . G G G G G . G G G G G G G . G G G G G G G G G . . . G . . . . . G . . . . . G . . . G G . . . . . . . . . . . . . . .    
153	T T T T T T T T T T T T T T T . T T T T T . T T T T T T T . T T T T T T T T T . . . T . . . . . T . . . . . T . . . T T . . . . . . . . . . . . . . .    
154	A A A A A A A A A A A A A A A . A A A A A . A A A A A A A . A A A A A A A A A . . . A . . . . . A . . . . . A . . . A A . . . . . . . . . . . . . . .    
155	T T T T T T T T T T T T T T T . T T T T T . T T T T T T T . T T T T T T T T T . . . T . . . . . T . . . . . T . . . T T . . . . . . . . . . . . . . .    
156	G G G G G G G G G G G G G G G . G G G G G . G G G G G G G . G G G G G G G G G . . . G . . . . . G . . . . . G . . . G G . . . . . . . . . . . . . . .    
157	T T T T T T T T T T T T T T T . T T T T T . T T T T T T T . T T T T T T T T T . . . T . . . . . T . . . . . T . . . T T . . . . . . . . . . . . . . .    
158	G G G G G G G G G G G G G G G . G G G G G . G G G G G G G . G G G G G G G G G . . . G . . . . . G . . . . . G . . . G G . . . . . . . . . . . . . . .    
159	T T T T T T T T T T T T T T T . T T T T T . T T T T T T T . T T T T T T T T T . . . T . . . . . T . . . . . T . . . T T . . . . . . . . . . . . . . .    
160	T T T T T T T T T T T T T T T . T T T T T . T T T T T T T . T T T T T T T T T . . . T . . . . . T . . . . . T . . . T T . . . . . . . . . . . . . . .    
161	C C C C C C C C C C C C C C C . C C C C C . C C C C C C C . C C C C C C C C C . . . C . . . . . C . . . . . C . . . C C . . . . . . . . . . . . . . .    
162	A A A A A A A A A A A A A A A . A A A A A . A A A A A A A . A A A A A A A A A . . . A . . . . . A . . . . . A . . . A A . . . . . . . . . . . . . . .    
163	A A A A A A A A A A A A A A A . A A A A A . A A A A A A A . A A A A A A A A A . . . A . A . . . A . . . . . A . . . A A . . . . . . . . . . . . . . .    
164	A A A A A A A A A A A A A A A . A A A A A . A A A A A A A . A A A A A A A A A . . . A . A . . . A . . . . . A . . . A A . . . . . . . . . . . . . . .    
165	A A A A A A A A A A A A A A A . A A A A A . A A A A A A A . A A A A A A A A A . . . A . A . . . A . . . . . A . . . A A . . . . . . . . . . . . . . .    
166	T T T T T T T T T T T T T T T . T T T T T . T T T T T T T . T T T T T T T T T . . . T . T . . . T . . . . . T . . . T T . . . . . . . . . . . . . . .    
167	G G G G G G G G G G G G G G G . G G G G G . G G G G G G G . G G G G G G G G G . . . G . G . . . G . . . . . G . . . G G . . . . . . . . . . . . . . .    
168	C C C C C C C C C C C C C C C . C C C C C . C C C C C C C . C C C C C C C C C . . . C . C . . . C . . . . . C . . . C C . . . . . . . . . . . . . . .    
169	A A A A A A A A A A A A A A A . A A A A A . A A A A A A A . A A A A A A A A A . . . A . A . . . A . . . . . A . . . A A . . . . . . . . . . . . . . .    
170	C C C C C C C C C C C C C C C . C C C C C . C C C C C C C . C C C C C C C C C . . . C . C . . . C . . . . . C . . . C C . . . . . . . . . . . . . . .    
171	A A A A A A A A A A A A A A A . A A A A A . A A A A A A A . A A A A A A A A A . . . A . A . . . A . . . . . A . . . A A . . . . . . . . . . . . . . .    
172	C C C C C C C C C C C C C C C . C C C C C . C C C C C C C . C C C C C C C C C . . . C . C . . . C . . . . . C . . . C C . . . . . . . . . . . . . . .    
173	C C C C C C C C C C C C C C C . C C C C C . C C C C C C C . C C C C C C C C C . . . C . C . . . C . . . . . C . . . C C . . . . . . . . . . . . . . .    
174	A A A A A A A A A A A A A A A . A A A A A . A A A A A A A . A A A A A A A A A . . . A . A . . . A . . . . . A . . . A A . . . . . . . . . . . . . . .    
175	A A A A A A A A A A A A A A A . A A A A A . A A A A A A A . A A A A A A A A A . . . A . A . . . A . . . . . A . . . A A . . . . . . . . . . . . . . .    
176	A A A A A A A A A A A A A A A . A A A A A . A A A A A A A . A A A A A A A A A . . . A . A . . . A . . . . . A . . . A A . . . . . . . . . . . . . . .    
177	G G G G G G G G G G G G G G G . G G G G G . G G G G G G G . G G G G G G G G G . . . G . G . . . G . . . . . G . . . G G . . . . . . . . . . . . . . .    
178	A A A A A A A A A A A A A A A . A A A A A . A A A A A A A . A A A A A A A A A . . . A . A . . . A . . . . . A . . . A A A A . . . . . . . . . . . . .    
179	G G G G G G G G G G G G G G G . G G G G G . G G G G G G G . G G G G G G G G G . . . G . G . . . G . . . . . G . . . G G G G . . . . . . . . . . . . .    
180	A A A A A A A A A A A A A A A . A A A A A . A A A A A A A . A A A A A A A A A . . . A . A . . . A . . . . . A . . . A A A A . . . . . . . . . . . . .    
181	C C C C C C C C C C C C C C C . C C C C C . C C C C C C C . C C C C C C C C C . . . C . C . . . C . . . . . C . . . C C C C . . . . . . . . . . . . .    
182	A A A A A A A A A A A A A A A . A A A A A . A A A A A A A . A A A A A A A A A . . . A . A . . . A . . . . . A . . . A A A A . . . . . . . . . . . . .    
183	T T T T T T T T T T T T T T T T T T T T T . T T T T T T T . T T T T T T T T T . . . T . T . . . T . . . . . T . . . T T T T . . . . . . . . . . . . .    
184	A A A A A A A A A A A A A A A A A A A A A . A A A A A A A . A A A A A A A A A . . . A . A . . . A . . . . . A . . . A A A A . . . . . . . . . . . . .    
185	C C C C C C C C C C C C C C C C C C C C C . C C C C C C C . C C C C C C C C C . . . C . C . . . C . . . . . C . . . C C C C . . . . . . . . . . . . .    
186	C C C C C C C C C C C C C C C C C C C C C . C C C C C C C . C C C C C C C C C . . . C . C . . . C . . . . . C . . . C C C C . . . . . . . . . . . . .    
187	A A A A A A A A A A A A A A A A A A A A A . A A A A A A A . A A A A A A A A A . . . A . A . . . A . . . . . A . . . A A A A . A . . . . . . . . . . .    
188	A A A A A A A A A A A A A A A A A A A A A . A A A A A A A . A A A A A A A A A . . . A . A . . . A . . . . . A . . . A A A A . A . . . . . . . . . . .    
189	A A A A A A A A A A A A A A A A A A A A A . A A A A A A A . A A A A A A A A A . . . A . A . . . A . . . . . A . . . A A A A . A . . . . . . . . . . .    
190	A A A A A A A A A A A A A A A A A A A A A . A A A A A A A . A A A A A A A A A . . . A . A . . . A . . . . . A . . . A A A A . A . . . . . . . . . . .    
191	C C C C C C C C C C C C C C C C C C C C C . C C C C C C C . C C C C C C C C C . . . C . C . . . C . . . . . C . . . C C C C . C . . . . . . . . . . .    
192	T T T T T T T T T T T T T T T T T T T T T . T T T T T T T . T T T T T T T T T . . . T . T . . . T . . . . . T . . . T T T T . T . . . . . . . . . . .    
193	A A A A A A A A A A A A A A A A A A A A A A A A A A A A A . A A A A A A A A A . . . A . A . . . A . . . . . A . . . A A A A . A . . . . . . . . . . .    
194	A A A A A A A A A A A A A A A A A A A A A A A A A A A A A . A A A A A A A A A . . . A . A . . . A . . . . . A . . . A A A A . A . . . . . . . . . . .    
195	A A A A A A A A A A A A A A A A A A A A A A A A A A A A A . A A A A A A A A A . . . A . A . . . A . . . . . A . . . A A A A . A . . . . . . . . . . .    
196	A A A A A A A A A A A A A A A A A A A A A A A A A A A A A . A A A A A A A A A . . . A . A . . . A . . . . . A . . . A A A A . A . . . . . . . . . . .    
197	T T T T T T T T T T T T T T T T T T T T T T T T T T T T T . T T T T T T T T T . . . T . T . . . T . . . . . T . . . A T T T . T . . . . . . . . . . .    
198	A A A A A A A A A A A A A A A A A A A A A A A A A A A A A . A A A A A A A A A . . . A . A . . . A . . . . . A . . . A A A A . A . . . . . . . . . . .    
199	A A A A A A A A A A A A A A A A A A A A A A A A A A A A A . A A A A A A A A A . . . A . A . . . A . . . . . A . . . A A A A . A . . A . . . . . . . .    
200	A A A A A A A A A A A A A A A A A A A A A A A A A A A A A . A A A A A A A A A . . . A . A . . . A . . . . . A . . . A A A A . A . . A . . . . . . . .    
201	A A A A A A A A A A A A A A A A A A A A A A A A A A A A A . A A A A A A A A A . . . A . A . . . A . . . . . A . . . A A A A . A . . A . . . . . . . .    
202	C C C C C C C C C C C C C C C C C C C C C C C C C C C C C . C C C C C C C C C . . . C . C . . . C . . . . . C . . . C C C C . C . . C . . . . C . . .    
203	A A A A A A A A A A A A A A A A A A A A A A A A A A A A A . A A A A A A A A A . . . A . A . . A A A A . . . A . . . A A A A . A . . A . . . . A . . .    
204	A A A A A A A A A A A A A A A A A A A A A A A A A A A A A . A A A A A A A A A . . . A . A . . A A A A . . . A . . . A A A A . A . . A A . . . A . . .    
205	A A A A A A A A A A A A A A A A A A A A A A A A A A A A A . A A A A A A A A A . . . A . A . . A A A A . . . A . . . A A A A . A . . A A . . . A . . .    
206	A A A A A A A A A A A A A A A A A A A A A A A A A A A A A . A A A A A A A A A . . . A . A . . A A A A . . . A . . . A A A A . A . . A A . . . A . . .    
207	A A A A A A A A A A A A A A A A A A A A A A A A A A A A A . A A A A A A A A A . . . A . A . . A A A A . . . A . . . A A A A . A . . A A . . . A . . .    
208	G G G G G G G G G G G G G G G G G G G G G G G G G G G G G . G G G G G G G G G . . . G . G . . G G G G . . . G . . . G G G G . G . . G G . . . G . . .    
209	A A A A A A A A A A A A A A A A A A A A A A A A A A A A A . A A A A A A A A A . . . A . A . . A A A A . . . A . . . A A A A . A . . A A . . . A . . .    
210	C C C C C C C C C C C C C C C C C C C C C C C C C C C C C . C C C C C C C C C . . . C . C . . C C C C . . . C . . . C C C C . C . . C C . . . C . . .    
211	T T T T T T T T T T T T T T T T T T T T T T T T T T T T T . T T T T T T T T T . . . T . T . . T T T T . . . T . . . T T T T . T . . T T . . . T . . .    
212	G G G G G G G G G G G G G G G G G G G G G G G G G G G G G . G G G G G G G G G . . . G . G . . G G G G . . . G . . . G G G G . G . . G G . . . G . . .    
213	T T T T T T T T T T T T T T T T T T T T T T T T T T T T T . T T T T T T T T T . . . T . T . . T T T T . . T T . . . T T T T . T . . T T . . . T . . .    
214	C C C C C C C C C C C C C C C C C C C C C C C C C C C C C . C C C C C C C C C . . . C . C . . C C C C . . C C . . . C C C C . C . . C C . . . C . . .    
215	A A A A A A A A A A A A A A A A A A A A A A A A A A A A A . A A A A A A A A A . . . A . A . . A A A A . . A A . . . A A A A . A . . A A . . . A . . .    
216	A A A A A A A A A A A A A A A A A A A A A A A A A A A A A . A A A A A A A A A . . . A . A . . A A A A . . A A . . . A A A A . A . . A A . . . A . . .    
217	A A A A A A A A A A A A A A A A A A A A A A A A A A A A A . A A A A A A A A A . . . A . A . . A A A A . . A A . . . A A A A . A . . A A . . . A . . .    
218	G G G G G G G G G G G G G G G G G G G G G G G G G G G G G . G G G G G G G G G . . . G . G . . G G G G . . G G . . . G G G G . G . . G G . . . G . . .    
219	C C C C C C C C C C C C C C C C C C C C C C C C C C C C C . C C C C C C C C C . . . C . C . . C C C C . . C C . . . C C C C . C . . C C . . . C . . .    
220	C C C C C C C C C C C C C C C C C C C C C C C C C C C C C . C C C C C C C C C . . . C . C . . C C C C . . C C . . . C C C C . C . . C C . . . C . . .    
221	T T T T T T T T T T T T T T T T T T T T T T T T T T T T T . T T T T T T T T T . . . T . T . . T T T T . . T T . . . T T T T . T . . T T . . . T . . .    
222	G G G G G G G G G G G G G G G G G G G G G G G G G G G G G . G G G G G G G G G . . . G . G . . G G G G . . G G . . . G G G G . G . . G G . . . G . . .    
223	T T T T T T T T T T T T T T T T T T T T T T T T T T T T T . T T T T T T T T T . . . T . T . . T T T T . . T T . . . T T T T . T . . T T . . . T . . .    
224	A A A A A A A A A A A A A A A A A A A A A A A A A A A A A . A A A A A A A A A . . . A . A . . A A A A . . A A . . . A A A A . A . . A A . . . A . . .    
225	A A A A A A A A A A A A A A A A A A A A A A A A A A A A A . A A A A A A A A A . . . A . A . . A A A A . . A A . . . A A A A . A . . A A . . . A . . .    
226	A A A A A A A A A A A A A A A A A A A A A A A A A A A A A . A A A A A A A A A . . . A . A . . A A A A . . A A . . . A A A A . A . . A A . . . A . . .    
227	A A A A A A A A A A A A A A A A A A A A A A A A A A A A A A A A A A A A A A A . . . A . A . . A A A A . . A A . . . A A A A . A . . A A . . . A . . .    
228	T T T T T T T T T T T T T T T T T T T T T T T T T T T T T T T T T T T T T T T . . . T . T . . T T T T . . T T . . . T T T T . T . . T T . . . T . T .    
229	G G G G G G G G G G G G G G G G G G G G G G G G G G G G G G G G G G G G G G G . . . G . G . . G G G G . . G G . . . G G G G . G . . G G . . . G . G .    
230	C C C C C C C C C C C C C C C C C C C C C C C C C C C C C C C C C C C C C C C . . . C . C . . C C C C . . C C . . . C C C C . C . . C C . . . C . C .    
231	A A A A A A A A A A A A A A A A A A A A A A A A A A A A A A A A A A A A A A A . . . A . A . . A A A A . . A A . . . A A A A . A . . A A . . . A . A .    
232	A A A A A A A A A A A A A A A A A A A A A A A A A A A A A A A A A A A A A A A . . . A . C . . A A A A . . A A . . . A A A A . A . . A A . . . A . A .    
233	C C C C C C C C C C C C C C C C C C C C C C C C C C C C C C C C C C C C C C C . . . C . C . . C C C C . . C C . . . C C C C . C . . C C . . . C . C .    
234	C C C C C C C C C C C C C C C C C C C C C C C C C C C C C C C C C C C C C C C . . . C . C . . C C C C . . C C . . . C C C C . C . C C C . . . C . C .    
235	T T T T T T T T T T T T T T T T C T T T T T T T T T T T T T T T T T T T T T T . . . T . T . . T T T T . . T T . . . T T T T . T . T T T . . . T . T .    
236	C C C C C C C C C C C C C C C C C C C C C C C C C C C C C C C C C C C C C C C . . . C . C . . C C C C . . C C . . . C C C C . C . C C C . . C C . C .    
237	C C C C C C C C C C C C C C C C C C C C C C C C C C C C C C C C C C C C C C C . . . C . C . . C C C C . . C C . . . C C C C . C . C C C . . C C . C .    
238	G G G G G G G G G G G G G G G G G G G G G G G G G G G G G G G G G G G G G G G . . . G . G . . G G G G . . G G G . . G G G G . G . G G G . . G G . G .    
239	T T T T T T T T T T T T T T T T T T T T T T T T T T T T T T T T T T T T T T T . . . T . T . . T T T T . . T T T . . T T T T . T . T T T . . T T . T .    
240	C C C C C C C C C C C C C C C C C C C C C C C C C C C C C C C C C C C C C C C . . . C . C . . C C C C . C C C C . . C C C C . C . C C C . . C C . C .    
241	A A A A A A A A A A A A A A A A A A A A A A A A A A A A A A A A A A A A A A A . . . A . A . . A A A A . A A A A . . A A A A . A . A A A . . A A . A .    
242	C C C C C C C C C C C C C C C C C C C C C C C C C C C C C C C C C C C C C C C . . . C C C . . C C C C . C C C C C . C C C C . C . C C C . . C C . C .    
243	A A A A A A A A A A A A A A A A A A A A A A A A A A A A A A A A A A A A A A A . . . A A A . . A A A A . A A A A A . A A A A . A . A A A . . A A . A .    
244	T T T T T T T T T T T T T T T T T T T T T T T T T T T T T T T T T T T T T T T . . . T T T . . T T T T . T T T T T . T T T T . T . T T T . . T T . T .    
245	A A A A A A A A A A A A A A A A A A A A A A A A A A A A A A A A A A A A A A A . . . A A A . . A A A A . A A A A A . A A A A . A . A A A . . A A . A .    
246	G G G G G G G G G G G G G G G G G G G G G G G G G G G G G G G G G G G G G G G . . . G G G . . G G G G . G G G G G . G G G G . G . G G G . . G G . G .    
247	C C C C C C C C C C C C C C C C C C C C C C C C C C C C C C C C C C C C C C C . . . C C C . . C C C C . C C C C C . C C C C . C . C C C . . C C . C .    
248	A A A A A A A A A A A A A A A A A A A A A A A A A A A A A A A A A A A A A A A . . . A A A . . A A A A . A A A A A . A A A A . A . A A A . . A A . A .    
249	A A A A A A A A A A A A A A A A A A A A A A A A A A A A A A A A A A A A A A A . . . A A A . . A A A A . A A A A A . A A A A . A . A A A . . A A . A .    
250	T T T T T T T T T T T T T T T T T T T T T T T T T T T T T T T T T T T T T T T . . . T T T . . T T T T . T T T T T . T T T T . T . T T T . . T T . T .    
251	C C C C C C C C C C C C C C C C C C C C C C C C C C C C C C C C C C C C C C C . . . C C C . . C C C C . C C C C C . C C C C . C . C C C . . C C . C .    
252	A A A A A A A A A A A A A A A A A A A A A A A A A A A A A A A A A A A A A A A . . . A A A . . A A A A . A A A A A A A A A A . A . A A A . . A A . A .    
253	G G G G G G G G G G G G G G G G G G G G G G G G G G G G G G G G G G G G G G G . . . G G G G . G G G G . G G G G G G G G G G . G . G G G . . G G . G .    
254	T T T T T T T T T T T T T T T T T T T T T T T T T T T T T T T T T T T T T T T . . . T T T T . T T T T . T T T T T T T T T T . T . T T T . . T T . T .    
255	A A A A A A A A A A A A A A A A A A A A A A A A A A A A A A A A A A A A A A A . . . A A A A . A A A A . A A A A A A A A A A . A . A A A . . A A . A .    
256	G G G G G G G G A G A G G G G G A G G G G G G G G G G G G G G G G G G G G G G . . . G G G G . G G G G . G G G G G G G G G G . G . G G G . . G G . G .    
257	A A A A A A A A A A A A A A A A A A A A A A A A A A A A A A A A A A A A A A A . . . A A A A . A A A A . A A A A A A A A A A . A A A A A . . A A . A .    
258	A A A A A A A A A A A A A A A A A A A A A A A A A A A A A A A A A A A A A A A . . . A A A A . A A A A . A A A A A A A A A A . A A A A A . . A A . A .    
259	A A A A A A A A A A A A A A A A A A A A A A A A A A A A A A A A A A A A A A A . . . A A A A . A A A A . A A A A A A A A A A . A A A A A . . A A . A .    
260	A A A A A A A A A A A A A A A A A A A A A A A A A A A A A A A A A A A A A A A . . . A A A A . A A A A . A A A A A A A A A A . A A A A A . . A A . A .    
261	A A A A A A A A A A A A A A A A A A A A A A A A A A A A A A A A A A A A A A A . . . A A A A . A A A A . A A A A A A A A A A . A A A A A . . A A . A .    
262	T T T T T T T T T T T T T T T T T T T T T T T T T T T T T T T T T T T T T T T . . . T T T T . T T T T . T T T T T T T T T T . T T T T T . . T T . T .    
263	G G G G G G G G G G G G G G G G G G G G G G G G G G G G G G G G G G G G G G G . . . G G G G . G G G G . G G G G G G G G G G . G G G G G . . G G . G .    
264	C C C C C C C C C C C C C C C C C C C C C C C C C C C C C C C C C C C C C C C . . . C C C C . C C C C . C C C C C C C C C C . C C C C C . . C C . C .    
265	C C C C C C C C C C C C C C C C C C C C C C C C C C C C C C C C C C C C C C C . . . C C C C . C C C C . C C C C C C C C C C . C C C C C . . C C . C .    
266	C C C C C C C C C C C C C C C C C C C C C C C C C C C C C C C C C C C C C C C . . . C C C C . C C C C . C C C C C C C C C C . C C C C C . . C C . C .    
267	A A A A A A A A A A A A A A A A A A A A A A A A A A A A A A A A A A A A A A A . . . A A A A . A A A A . A A A A A A A A A A . A A A A A . . A A . A .    
268	T T T T T T T T T T T T T T T T T T T T T T T T T T T T T T T T T T T T T T T . . . T T T T . T T T T . T T T T T T T T T T . T T T T T . . T T . T .    
269	A A A A A A A A A A A A A A A A A A A A A A A A A A A A A A A A A A A A A A A . . . A A A A . A A A A . A A A A A A A A A A . A A A A A . . A A . A .    
270	T T T T T T T T T T T T T T T T T T T T T T T T T T T T T T T T T T T T T T T . . . T T T T . T T T T . T T T T T T T T T T . T T T T T . . T T . T .    
271	G G G G G G G G G G G G G G G G G G G G G G G G G G G G G G G G G G G G G G G . . . G G G G . G G G G . G G G G G G G G G G . G G G G G . . G G . G .    
272	C C C C C C C C C C C C C C C C C C C C C C C C C C C C C C C C C C C C C C C . . . C C C C . C C C C . C C C C C C C C C C . C C C C C . . C C . C .    
273	T T T T T T T T T T T T T T T T T T T T T T T T T T T T T T T T T T T T T T T . . . T T T T . T T T T . T T T T T T T T T T . T T T T T . . T T . T .    
274	G G G G G G G G G G G G G G G G G G G G G G G G G G G G G G G G G G G G G G G . . . G G G G . G G G G . G G G G G G G G G G . G G G G G . . G G . G .    
275	G G G G G G G G G G G G G G G G G G T G G G G G G G G G G G G G G G G G G G G . . . G G G G . G G G G . G G G G T G G G G G . G G G G G . . G G . G .    
276	G G G G G G G G G G G G G G G G G G G G G G G G G G G G G G G G G G G G G G G . . . G G G G . G G G G . G G G G G G G G G G . G G G G G . . G G . G .    
277	G G G G G G G G G G G G G G G G G G G G G G G G G G G G G G G G G G G G G G G . . . G G G G . G G G G . G G G G G G G G G G . G G G G G . . G G . G .    
278	- - - - - - - - - - - - - - - - - - - - - - - - - - - - - - - - - - - - - - - . . . - - - - . - - - - . - G - - - - - - - - . - - - - - . . - - . - .    
279	A A A A A A A A A A A A A A A A A A A A A A A A A A A A A A A A A A A A A A A . . . A A A A . A A A A . A A A A A A A A A A . A A A A A . . A A . A .    
280	T T T T T T T T T T T T T T T T T T T T T T T T T T T T T T T T T T T T T T T . . . T T T T . T T T T . T T T T T T T T T T . T T T T T . . T T . T .    
281	T T T T T T T T T T T T T T T T T T T T T T T T T T T T T T T T T T T T T T T . . . T T T T . T T T T . T T T T T T T T T T . T T T T T . . T T . T .    
282	A A A A A A A A A A A A A A A A A A A A A A A A A A A A A A A A A A A A A A A . . . A A A A . A A A A . A A A A A A A A A A . A A A A A . . A A . A .    
283	T T T T T T T T T T T T T T T T T T T T T T T T T T T T T T T T T T T T T T T . . . T T T T . T T T T . T T T T T T T T T T . T T T T T . . T T . T .    
284	C C C C C C C C C C C C C C C C C C C C C C C C C C C C C C C C C C C C C C C . . . C C C C . C C C C . C C C C C C C C C C . C C C C C . . C C . C .    
285	G G G G G G G G G G A G G G G G G A A G G G G G G G A G G G G G G G G G G G G . . . G A G G . G G G G . G G G A A A G G G G . G G G G G . . G G . G .  8	 transition  
286	G G G G G G G G G G A G G G G G G G G G G G G G G G G G G G G G G G G G G G G . . . G G G G . G G G G G G G G G G G G G G G . G G G G G . . G G . G .    
287	A A A A A A A A A A A A A A A A A A A A A A A A A A A A A A A A A A A A A A A . . . A A A A . A A A A A A A A A A A A A A A . A A A A A . . A A . A .    
288	A A A A A A A A A A A A A A A A A A A A A A A A A A A A A A A A A A A A A A A . . . A A A A . A A A A A A A A A A A A A A A . A A A A A . . A A . A .    
289	G G G G G G G G G G - G G G G G G - - G G G G G G G - G G G G G G G G G G G G . . . G A G G . G G G G G G G G A A A G G G G . G G G G G . . G G . G .    
290	A A A A A A A A A A A A A A A A A A A A A A A A A A A A A A A A A A A A A A A . . . A A A A . A A A A A A A A A A A A A A A . A A A A A . . A A . A .    
291	A A A A A A A A A A A A A A A A A A A A A A A A A A A A A A A A A A A A A A A . . . A A A A . A A A A A A A A A A A A A A A . A A A A A . . A A . A .    
292	A A A A A A A A A A A A A A A A A A A A A A A A A A A A A A A A A A A A A A A . . . A A A A . A A A A A A A A A A A A A A A . A A A A A . . A A . A A    
293	A A A A A A A A A A A A A A A A A A A A A A A A A A A A A A A A A A A A A A A . . . A A A A . A A A A A A A A A A A A A A A . A A A A A . . A A . A A    
294	A A A A A A A A A A A A A A A A A A A A A A A A A A A A A A A A A A A A A A A . . . A A A A . A A A A A A A A A A A A A A A . A A A A A . . A A . A A    
295	A A A A A A A A A A A A A A A A A A A A A A A A A A A A A A A A A A A A A A A . . . A A A A . A A A A A A A A A A A A A A A . A A A A A . . A A . A A    
296	A A A A A A A A A A A A A A A A A A A A A A A A A A A A A A A A A A A A A A A . . . A A A A . A A A A A A A A A A A A A A A . A A A A A . . A A . A A    
297	A A A A A A A A A A A A A A A A A A A A A A A A A A A A A A A - A A A A A A A . . . A - A A . A A A A A A A A A A A A A A A . A A A A A . . A A . A A    
298	A A A A A A A A A A A A - - A A A A A A A A A A A A A A A A - - A A A A A - - . . . A - - - . - A A A A A A A - - - A A A A . A A A A A . . A - . A A  14	 indel  
299	- - - A - - - - - - - - - - - - - - - - - - - - - - - - A A - - - - - - - - - . . . - - - - . - - - - - - - - - - - - - - - . - A - - - . . - - . - A    
300	T T T T T T T T T T T T T T T T T T T T T T T T T T T T T T T T T T T T T T T . . . T T T T . T T T T T T T T T T T T T T T . T T T T T . . T T . T T    
301	T T T T T T T T T T T T T T T T T T T T T T T T T T T T T T T T T T T T T T T . . . T T T T . T T T T T T T T T T T T T T T . T T T T T . . T T . T T    
302	C C C C C C C C C C C C C C C C C C C C C C C C C C C C C C C C C C C C C C C . . . C C C C . C C C C C C C C C C C C C C C . C C C C C . . C C . C C    
303	A A A A A A A A A A A A A A A A A A A A A A A A A A A A A A A A A A A A A A A . . . A A A A . A A A A A A A A A A A A A A A . A A A A A . . A A . A A    
304	C C C C C C C C C C C C C C C C C C C C C C C C C C C C C C C C C C C C C C C . . . C C C C . C C C C C C C C C C C C C C C . C C C C C . . C C . C C    
305	C C C C C C C C C C C C C C C C C C C C C C C C C C C C C C C C C C C C C C C . . . C C C C . C C C C C C C C C C C C C C C . C C C C C . . C C . C C    
306	C C C C C C C C C C C C C C C C C C C C C C C C C C C C C C C C C C C C C C C . . . C C C C . C C C C C C C C C C C C C C C . C C C C C . . C C . C C    
307	T T T T T T T T T T T T T T T T T T T T T T T T T T T T T T T T T T T T T T T . . . T T T T . T T T T T T T T T T T T T T T . T T T T T . . T T . T T    
308	A A A A A A A A A A A A A A A A A A A A A A A A A A A A A A A A A A A A A A A . . . A A A A . A A A A A A A A A A A A A A A . A A A A A . . A A . A A    
309	A A A A A A A A A A A A A A A A A A A A A A A A A A A A A A A A A A A A A A A . . . A A A A . A A A A A A A A A A A A A A A . A A A A A . . A A . A A    
310	T T T T T T T T T T T T T T T T T T T T T T T T T T T T T T T T T T T T T T T . . . T T T T . T T T T T T T T T T T T T T T . T T T T T T . T T . T T    
311	A A A A A A A A A A A A A A A A A A A A A A A A A A A A A A A A A A A A A A A . . . A A A A . A A A A A A A A A A A A A A A . A A A A A A . A A . A A    
312	T T T T T T T T T T T T T T T T T T T T T T T T T T T T T T T T T T T T T T T . . . T T T T . T T T T T T T T T T T T T T T . T T T T T T . T T . T T    
313	A A A A A A A A A A A A A A A A A A A A A A A A A A A A A A A A A A A A A A A . . . A A A A . A A A A A A A A A A A A A A A . A A A A A A . A A . A A    
314	C C C C C C C C C C C C C C C C C C C C C C C C C C C C C C C C C C C C C C C . . . C C C C . C C C C C C C C C C C C C C C . C C C C C C . C C . C C    
315	T T T T T T T T T T T T T T T T T T T T T T T T T T T T T T T T T T T T T T T . . . T T T T . T T T T T T T T T T T T T T T . T T T T T T . T T . T T    
316	T T T T T T T T T T T T T T T T T T T T T T T T T T T T T T T T T T T T T T T . . . T T T T . T T T T T T T T T T T T T T T . T T T T T T . T T . T T    
317	G G G G G G G G G G G G G G G G G G G G G G G G G G G G G G G G G G G G G G G . . . G G G G G G G G G G G G G G G G G G G G . G G G G G G . G G . G G    
318	A A A A A A A A A A A A A A A A A A A A A A A A A A A A A A A A A A A A A A A . . . A A A A A A A A A A A A A A A A A A A A . A A A A A A . A A . A A    
319	T T T T T T T T T T T T T T T T T T T T T T T T T T T T T T T T T T T T T T T . . . T T T T T T T T T T T T T T T T T T T T . T T T T T T . T T . T T    
320	A A A A A A A A A A A A A A A A A A A A A A A A A A A A A A A A A A A A A A A . . . A A A A A A A A A A A A A A A A A A A A . A A A A A A . A A . A A    
321	A A A A A A A A A A A A A A A A A A A A A A A A A A A A A A A A A A A A A A A . . . A A A A A A A A A A A A A A A A A A A A . A A A A A A . A A . A A    
322	C C C C C C C C C C C C C C C C C C C C C C C C C C C C C C C C C C C C C C C . . . C C C C C C C C C C C C C C C C C C C C . C C C C C C . C C . C C    
323	C C C C C C C C C C C C C C C C C C C C C C C C C C C C C C C C C C C C C C C . . . C C C C C C C C C C C C C C C C C C C C . C C C C C C . C C . C C    
324	A A A A A A A A A A A A A A A A A A A A A A A A A A A A A A A A A A A A A A A . . . A A A A A A A A A A A A A A A A A A A A . A A A A A A . A A . A A    
325	A A A A A A A A A A A A A A A A A A A A A A A A A A A A A A A A A A A A A A A . . . A A A A A A A A A A A A A A A A A A A A . A A A A A A . A A . A A    
326	G G G G G G G G G G G G G G G G G G G G G G G G G G G G G G G G G G G G G G G . . . G G G G G G G G G G G G G G G G G G G G . G G G G G G . G G . G G    
327	A A A A A A A A A A A A A A A A A A A A A A A A A A A A A A A A A A A A A A A . . . A A A A A A A A A A A A A A A A A A A A . A A A A A A . A A . A A    
328	T T T T T T T T T T T T T T T T T T T T T T T T T T T T T T T T T T T T T T T . . . T T T T T T T T T T T T T T T T T T T T . T T T T T T . T T . T T    
329	A A A A A A A A A A A A A A A A A A A A A A A A A A A A A A A A A A A A A A A . . . A A A A A A A A A A A A A A A A A A A A . A A A A A A . A A . A A    
330	T T T T T T T T T T T T T T T T T T T T T T T T T T T T T T T T T T T T T T T . . . T T T T T T T T T T T T T T T T T T T T . T T T T T T . T T . T T    
331	T T T T T T T T T T T T T T T T T T T T T T T T T T T T T T T T T T T T T T T . . . T T T T T T T T T T T T T T T T T T T T . T T T T T T . T T . T T    
332	A A A A A A A A A A A A A A A A A A A A A A A A A A A A A A A A A A A A A A A . . . A A A A A A A A A A A A A A A A A A A A . A A A A A A . A A . A A    
333	C C C C C C C C C C C C C C C C C C C C C C C C C C C C C C C C C C C C C C C . . . C C C C C C C C C C C C C C C C C C C C . C C C C C C . C C . C C    
334	C C C C C C C C C C C C C C C C C C C C C C C C C C C C C C C C C C C C C C C . . . C C C C C C C C C C C C C C C C C C C C . C C C C C C . C C . C C    
335	C C C C C C C C C C C C C C C C C C C C C C C C C C C C C C C C C C C C C C C . . . C C C C C C C C C C C C C C C C C C C C . C C C C C C . C C . C C    
336	A A A A A A A A A A A A A A A A A A A A A A A A A A A A A A A A A A A A A A A . . . A A A A A A A A A A A A A A A A A A A A . A A A A A A . A A . A A    
337	A A A A A A A A A A A A A A A A A A A A A A A A A A A A A A A A A A A A A A A . . . A A A A A A A A A A A A A A A A A A A A . A A A A A A . A A . A A    
338	T T T T T T T T T T T T T T T T T T T T T T T T T T T T T T T T T T T T T T T . . . T T T T T T T T T T T T T T T T T T T T T T T T T T T . T T . T T    
339	T T T T T T T T T T T T T T T T T T T T T T T T T T T T T T T T T T T T T T T . . . T T T T T T T T T T T T T T T T T T T T T T T T T T T . T T . T T    
340	A A A A A A A A A A A A A A A A A A A A A A A A A A A A A A A A A A A A A A A . . . A A A A A A A A A A A A A A A A A A A A A A A A A A A . A A . A A    
341	C C C C C C C C C C C C C C C C C C C C C C C C C C C C C C C C C C C C C C C . . . C C C C C C C C C C C C C C C C C C C C C C C C C C C . C C . C C    
342	A A A A A A A A A A A A A A A A A A A A A A A A A A A A A A A A A A A A A A A . . . A A A A A A A A A A A A A A A A A A A A A A A A A A A . A A . A A    
343	A A A A A A A A A A A A A A A A A A A A A A A A A A A A A A A A A A A A A A A . . . A A A A A A A A A A A A A A A A A A A A A A A A A A A . A A . A A    
344	A A A A A A A A A A A A A A A A A A A A A A A A A A A A A A A A A A A A A A A . . . A A A A A A A A A A A A A A A A A A A A A A A A A A A . A A . A A    
345	A A A A A A A A A A A A A A A A A A A A A A A A A A A A A A A A A A A A A A A . . . A A A A A A A A A A A A A A A A A A A A A A A A A A A . A A . A A    
346	C C C C C C C C C C C C C C C C C C C C C C C C C C C C C C C C C C C C C C C . . . C C C C C C C C C C C C C C C C C C C C C C C C C C C . C C C C C    
347	A A A A A A A A A A A A A A A A A A A A A A A A A A A A A A A A A A A A A A A . . . A A A A A A A A A A A A A A A A A A A A A A A A A A A . C A A A A    
348	C C C C C C C C C C C C C C C C C C C C C C C C C C C C C C C C C C C C C C C . . . C C C C C C C C C C C C C C C C C C C C C C C C C C C . C C C C C    
349	A A A A A A A A A A A A A A A A A A A A A A A A A A A A A A A A A A A A A A A . . . A A A A A A A A A A A A A A A A A A A A A A A A A A A . A A A A A    
350	A A A A A A A A A A A A A A A A A A A A A A A A A A A A A A A A A A A A A A A . . . A A A A A A A A A A A A A A A A A A A A A A A A A A A . A A A A A    
351	A A A A A A A A A A A A A A A A A A A A A A A A A A A A A A A A A A A A A A A . . . A A A A A A A A A A A A A A A A A A A A A A A A A A A . A A A A A    
352	T T T T T T T T T T T T T T T T T T T T T T T T T T T T T T T T T T T T T T T . . . T T T T T T T T T T T T T T T T T T T T T T T T T T T . T T T T T    
353	C C C C C C C C C C C C C C C C C C C C C C C C C C C C C C C C C C C C C C C . . . C C C C C C C C C C C C C C C C C C C C C C C C C C C . C C C C C    
354	G G G G G G G G G G G G G G G G G G G G G G G G G G G G G G G G G G G G G G G . . . G G G G G G G G G G G G G G G G G G G G G G G G G G G . G G G G G    
355	G G G G G G G G G G G G G G G G G G G G G G G G G G G G G G G G G G G G G G G . . . G G G G G G G G G G G G G G G G G G G G G G G G G G G . G G G G G    
356	T T T T T T T T T T T T T T T T T T T T T T T T T T T T T T T T T T T T T T T . . . T T T T T T T T T T T T T T T T T T T T T T T T T T T . T T T T T    
357	T T T T T T T T T T T T T T T T T T T T T T T T T T T T T T T T T T T T T T T . . . T T T T T T T T T T T T T T T T T T T T T T T T T T T . T T T T T    
358	A A A A A A A A A A A A A A A A A A A A A A A A A A A A A A A A A A A A A A A . . . A A A A A A A A A A A A A A A A A A A A A A A A A A A . A A A A A    
359	A A A A A A A A A A A A A A A A A A A A A A A A A A A A A A A A A A A A A A A . . . A A A A A A A A A A A A A A A A A A A A A A A A A A A . A A A A A    
360	C C C C C C C C C C C C C C C C C C C C C C C C C C C C C C C C C C C C C C C . . . C C C C C C C C C C C C C C C C C C C C C C C C C C C . C C C C C    
361	T T T T T T T T T T T T T T T T T T T T T T T T T T T T T T T T T T T T T T T . . . T T T T T T T T T T T T T T T T T T T T T T T T T T T . T T T T T    
362	T T T T T T T T T T T T T T T T T T T T T T T T T T T T T T T T T T T T T T T . . . T T T T T T T T T T T T T T T T T T T T T T T T T T T . T T T T T    
363	C C C C C C C C C C C C C C C C C C C C C C C C C C C C C C C C C C C C C C C . . . C C C C C C C C C C C C C C C C C C C C C C C C C C C . C C C C C    
364	A A A A A A A A A A A A A A A A A A A A A A A A A A A A A A A A A A A A A A A . . . A A A A A A A A A A A A A A A A A A A A A A A A A A A . A A A A A    
365	G G G G G G G G G G G G G G G G G G G G G G G G G G G G G G G G G G G G G G G . . . G G G G G G G G G G G G G G G G G G G G G G G G G G G . G G G G G    
366	C C C C C C C C C C C C C C C C C C C C C C C C C C C C C C C C C C C C C C C . . . C C C C C C C C C C C C C C C C C C C C C C C C C C C . C C C C C    
367	A A A A A A A A A A A A A A A A A A A A A A A A A A A A A A A A A A A A A A A . . . A A A A A A A A A A A A A A A A A A A A A A A A A A A . A A A A A    
368	A A A A A A A A A A A A A A A A A A A A A A A A A A A A A A A A A A A A A A A . . . A A A A A A A A A A A A A A A A A A A A A A A A A A A . A A A A A    
369	T T T T T T T T T T T T T T T T T T T T T T T T T T T T T T T T T T T T T T T . . . T T T T T T T T T T T T T T T T T T T T T T T T T T T . T T T T T    
370	T T T T T T T T T T T T T T T T T T T T T T T T T T T T T T T T T T T T T T T . . . T T T T T T T T T T T T T T T T T T T T T T T T T T T . T T T T T    
371	- - - - - - - - - - - - - - - - - - - - - - - - - - - - - - - - - - - - - - - . . . - - - - - - - - - - T - - - - - - - - - - - - - - - - . - - - - -    
372	G G G G G G G G G G G G G G G G G G G G G G G G G G G G G G G G G G G G G G G . . . G G G G G G G G G G G G G G G G G G G G G G G G G G G . G G G G G    
373	G G G G G G G G G G G G G G G G G G G G G G G G G G G G G G G G G G G G G G G . . . G G G G G G G G G G G G G G G G G G G G G G G G G G G . G G G G G    
374	A A A A A A A A A A A A A A A A A A A A A A A A A A A A A A A A A A A A A A A . . . A A A A A A A A A A A A A A A A A A A A A A A A A A A . A A A A A    
375	C C C C C C C C C C C C C C C C C C C C C C C C C C C C C C C C C C C C C C C . . . C C C C C C C C C C C C C C C C C C C C C C C C C C C . C C C C C    
376	A A A A A A A A A A A A A A A A A A A A A A A A A A A A A A A A A A A A A A A . . . A A A A A A A A A A A A A A A A A A A A A A A A A A A . A A A A A    
377	A A A A A A A A A A A A A A A A A A A A A A A A A A A A A A A A A A A A A A A . . . A A A A A A A A A A A A A A A A A A A A A A A A A A A . A A A A A    
378	A A A A A A A A A A A A A A A A A A A A A A A A A A A A A A A A A A A A A A A . . . A A A A A A A A A A A A A A A A A A A A A A A A A A A . A A A A A    
379	T T T T T T T T T T T T T T T T T T T T T T T T T T T T T T T T T T T T T T T . . . T T T T T T T T T T T T T T T T T T T T T T T T T T T . T T T T T    
380	T T T T T T T T T T T T T T T T T T T T T T T T T T T T T T T T T T T T T T T . . . T T T T T T T T T T T T T T T T T T T T T T T T T T T . T T T T T    
381	A A A A A A A A A A A A A A A A A A A A A A A A A A A A A A A A A A A A A A A . . . A A A A A A A A A A A A A A A A A A A A A A A A A A A . A A A A A    
382	A A A A A A A A A A A A A A A A A A A A A A A A A A A A A A A A A A A A A A A . . . A A A A A A A A A A A A A A A A A A A A A A A A A A A . A A A A A    
383	G G G G G G G G G G G G G G G G G G G G G G G G G G G G G G G G G G G G G G G . . . G G G G G G G G G G G G G G G G G G G G G G G G G G G . G G G G G    
384	A A A A A A A A A A A A A A A A A A A A A A A A A A A A A A A A A A A A A A A . . . A A A A A A A A A A A A A A A A A A A A A A A A A A A . A A A A A    
385	T T T T T T T T T T T T T T T T T T T T T T T T T T T T T T T T T T T T T T T . . . T T T T T T T T T T T T T T T T T T T T T T T T T T T . T T T T T    
386	G G G G G G G G G G G G G G G G G G G G G G G G G G G G G G G G G G G G G G G . . . G G G G G G G G G G G G G G G G G G G G G G G G G G G . G G G G G    
387	T T T T T T T T T T T T T T T T T T T T T T T T T T T T T T T T T T T T T T T . . . T T T T T T T T T T T T T T T T T T T T T T T T T T T . T T T T T    
388	A A A A A A A A A A A A A A A A A A A A A A A A A A A A A A A A A A A A A A A . . . A A A A A A A A A A A A A A A A A A A A A A A A A A A . A A A A A    
389	C C C C C C C C C C C C C C C C C C C C C C C C C C C C C C C C C C C C C C C . . . C C C C C C C C C C C C C C C C C C C C C C C C C C C . C C C C C    
390	A A A A A A A A A A A A A A A A A A A A A A A A A A A A A A A A A A A A A A A . . . A A A A A A A A A A A A A A A A A A A A A A A A A A A . A A A A A    
391	A A A A A A A A A A A A A A A A A A A A A A A A A A A A A A A A A A A A A A A . . . A A A A A A A A A A A A A A A A A A A A A A A A A A A . A A A A A    
392	A A A A A A A A A A A A A A A A A A A A A A A A A A A A A A A A A A A A A A A . . . A A A A A A A A A A A A A A A A A A A A A A A A A A A . A A A A A    
393	T T T T T T T T T T T T T T T T T T T T T T T T T T T T T T T T T T T T T T T . . . T T T T T T T T T T T T T T T T T T T T T T T T T T T . T T T T T    
394	G G G G G G G G G G G G G G G G G G G G G G G G G G G G G G G G G G G G G G G . . . G G G G G G G G G G G G G G G G G G G G G G G G G G G . G G G G G    
395	A A A A A A A A A A A A A A A A A A A A A A A A A A A A A A A A A A A A A A A . . . A A A A A A A A A A A A A A A A A A A A A A A A A A A . A A A A A    
396	T T T T T T T T T T T T T T T T T T T T T T T T T T T T T T T T T T T T T T T . . . T T T T T T T T T T T T T T T T T T T T T T T T T T T . T T T T T    
397	T T T T T T T T T T T T T T T T T T T T T T T T T T T T T T T T T T T T T T T . . . T T T T T T T T T T T T T T T T T T T T T T T T T T T . T T T T T    
398	T T T T T T T T T T T T T T T T T T T T T T T T T T T T T T T T T T T T T T T . . . T T T T T T T T T T T T T T T T T T T T T T T T T T T . T T T T T    
399	A A A A A A A A A A A A A A A A A A A A A A A A A A A A A A A A A A A A A A A . . . A A A A A A A A A A A A A A A A A A A A A A A A A A A . A A A A A    
400	G G G G G G G G G G C G G G G G G G G G G G G G G G G G G G G G G G G G G G G . . . G G G G G G G G G G G G G G G G G G G G G G G G G G G . G G G G G    
401	A A A A A A A A A A A A A A A A A A A A A A A A A A A A A A A A A A A A A A A . . . A A A A A A A A A A A A A A A A A A A A A A A A A A A . A A A A A    
402	A A A A A A A A A A A A A A A A A A A A A A A A A A A A A A A A A A A A A A A . . . A A A A A A A A A A A A A A A A A A A A A A A A A A A . A A A A A    
403	T T T T T T T T T T T T T T T T T T T T T T T T T T T T T T T T T T T T T T T . . . T T T T T T T T T T T T T T T T T T T T T T T T T T T . T T T T T    
404	T T T T T T T T T T T T T T T T T T T T T T T T T T T T T T T T T T T T T T T . . . T T T T T T T T T T T T T T T T T T T T T T T T T T T . T T T T T    
405	T T T T T T T T T T T T T T T T T T T T T T T T T T T T T T T T T T T T T T T . . . T T T T T T T T T T T T T T T T T T T T T T T T T T T . T T T T T    
406	A A A A A A A A A A A A A A A A A A A A A A A A A A A A A A A A A A A A A A A . . . A A A A A A A A A A A A A A A A A A A A A A A A A A A . A A A A A    
407	A A A A A A A A A A A A A A A A A A A A A A A A A A A A A A A A A A A A A A A . . . A A A A A A A A A A A A A A A A A A A A A A A A A A A . A A A A A    
408	A A A A A A A A A A A A A A A A A A A A A A A A A A A A A A A A A A A A A A A . . . A A A A A A A A A A A A A A A A A A A A A A A A A A A . A A A A A    
409	A A A A A A A A A A A A A A A A A A A A A A A A A A A A A A A A A A A A A A A . . . A A A A A A A A A A A A A A A A A A A A A A A A A A A . A A A A A    
410	A A A A A A A A A A A A A A A A A A A A A A A A A A A A A A A A A A A A A A A . . . A A A A A A A A A A A A A A A A A A A A A A A A A A A . A A A A A    
411	A A A A A A A A A A A A A A A A A A A A A A A A A A A A A A A A A A A A A A A . . . A A A A A A A A A A A A A A A A A A A A A A A A A A A . A A A A A    
412	T T T T T T T T T T T T T T T T T T T T T T T T T T T T T T T T T T T T T T T . . . T T T T T T T T T T T T T T T T T T T T T T T T T T T . T T T T T    
413	A A A A A A A A A A A A A A A A A A A A A A A A A A A A A A A A A A A A A A A . . . A A A A A A A A A A A A A A A A A A A A A A A A A A A . A A A A A    
414	A A A A A A A A A A A A A A A A A A A A A A A A A A A A A A A A A A A A A A A . . . A A A A A A A A A A A A A A A A A A A A A A A A A A A . A A A A A    
415	G G G G G G G G G G G G G G G G G G G G G G G G G G G G G G G G G G G G G G G . . . G G G G G G G G G G G G G G G G G G G G G G G G G G G . G G G G G    
416	C C C C C C C C C C C C C C C C C C C C C C C C C C C C C C C C C C C C C C C . . . C C C C C C C C C C C C C C C C C C C C C C C C C C C . C C C C C    
417	A A A A A A A A A A A A A A A A A A A A A A A A A A A A A A A A A A A A A A A . . . A A A A A A A A A A A A A A A A A A A A A A A A A A A . A A A A A    
418	A A A A A A A A A A A A A A A A A A A A A A A A A A A A A A A A A A A A A A A . . . A A A A A A A A A A A A A A A A A A A A A A A A A A A . A A A A A    
419	A A A A A A A A A A A A A A A A A A A A A A A A A A A A A A A A A A A A A A A . . . A A A A A A A A A A A A A A A A A A A A A A A A A A A . A A A A A    
420	A A A A A A A A A A A A A A A A A A A A A A A A A A A A A A A A A A A A A A A . . . A A A A A A A A A A A A A A A A A A A A A A A A A A A . A A A A A    
421	T T T T T T T T T T T T T T T T T T T T T T T T T T T T T T T T T T T T T T T . . . T T T T T T T T T T T T T T T T T T T T T T T T T T T . T T T T T    
422	A A A A A A A A A A A A A A A A A A A A A A A A A A A A A A A A A A A A A A A . . . A A A A A A A A A A A A A A A A A A A A A A A A A A A . A A A A A    
423	A A A A A A A A A A A A A A A A A A A A A A A A A A A A A A A A A A A A A A A A . . A A A A A A A A A A A A A A A A A A A A A A A A A A A . A A A A A    
424	A A A A A A A A A A A A A A A A A A A A A A A A A A A A A A A A A A A A A A A A . . A A A A A A A A A A A A A A A A A A A A A A A A A A A . A A A A A    
425	A A A A A A A A A A A A A A A A A A A A A A A A A A A A A A A A A A A A A A A A . . A A A A A A A A A A A A A A A A A A A A A A A A A A A . A A A A A    
426	T T T T T T T T T T T T T T T T T T T T T T T T T T T T T T T T T T T T T T T T . . T T T T T T T T T T T T T T T T T T T T T T T T T T T . T T T T T    
427	A A A A A A A A A A A A A A A A A A A A A A A A A A A A A A A A A A A A A A A A . . A A A A A A A A A A A A A A A A A A A A A A A A A A A . A A A A A    
428	A A A A A A A A A A A A A A A A A A A A A A A A A A A A A A A A A A A A A A A A . . A A A A A A A A A A A A A A A A A A A A A A A A A A A . A A A A A    
429	A A A A A A A A A A A A A A A A A A A A A A A A A A A A A A A A A A A A A A A A . . A A A A A A A A A A A A A A A A A A A A A A A A A A A . A A A A A    
430	A A A A A A A A A A A A A A A A A A A A A A A A A A A A A A A A A A A A A A A A . . A A A A A A A A A A A A A A A A A A A A A A A A A A A . A A A A A    
431	A A A A A A A A A A A A A A A A A A A A A A A A A A A A A A A A A A A A A A A A . . A A A A A A A A A A A A A A A A A A A A A A A A A A A . A A A A A    
432	A A A A A A A A A A A A A A A A A A A A A A A A A A A A A A A A A A A A A A A A . . A A A A A A A A A A A A A A A A A A A A A A A A A A A . A A A A A    
433	C C C C C C C C C C C C C C C C C C C C C C C C C C C C C C C C C C C C C C C C . . C C C C C C C C C C C C C C C C C C C C C C C C C C C . C C C C C    
434	A A A A A A A A A A A A A A A A A A A A A A A A A A A A A A A A A A A A A A A A . . A A A A A A A A A A A A A A A A A A A A A A A A A A A . A A A A A    
435	T T T T T T T T T T T T T T T T T T T T T T T T T T T T T T T T T T T T T T T T . T T T T T T T T T T T T T T T T T T T T T T T T T T T T . T T T T T    
436	T T T T T T T T T T T T T T T T T T T T T T T T T T T T T T T T T T T T T T T T . T T T T T T T T T T T T T T T T T T T T T T T T T T T T . T T T T T    
437	T T T T T T T T T T T T T T T T T T T T T T T T T T T T T T T T T T T T T T T T . T T T T T T T T T T T T T T T T T T T T T T T T T T T T . T T T T T    
438	C C C C C C C C C C C C C C C C C C C C C C C C C C C C C C C C C C C C C C C C . C C C C C C C C C C C C C C C C C C C C C C C C C C C C . C C C C C    
439	A A A A A A A A A A A A A A A A A A A A A A A A A A A A A A A A A A A A A A A A . A A A A A A A A A A A A A A A A A A A A A A A A A A A A . A A A A A    
440	G G G G G G G G G G G G G G G G G G G G G G G G G G G G G G G G G G G G G G G G . G G G G G G G G G G G G G G G G G G G G G G G G G G G G . G G G G G    
441	G G G G G G G G G G G G G G G G G G G G G G G G G G G G G G G G G G G G G G G G . G G G G G G G G G G G G G G G G G G G G G G G G G G G G . G G G G G    
442	A A A A A A A A A A A A A A A A A A A A A A A A A A A A A A A A A A A A A A A A . A A A A A A A A A A A A A A A A A A A A A A A A A A A A . A A A A A    
443	T T T T T T T T T T G T T T T T T G G T T T T T T T G T T T T T T T T T T T T T . T T G T T T T T T T T T T T G G G T T T T T T T T T T T . T T T T T  8	 transversion  
444	A A A A A A A A A A A A A A A A A A A A A A A A A A A A A A A A A A A A A A A A . A A A A A A A A A A A A A A A A A A A A A A A A A A A A . A A A A A    
445	C C C C C C C C C C C C C C C C C C C C C C C C C C C C C C C C C C C C C C C C . C C C C C C C C C C C C C C C C C C C C C C C C C C C C . C C C C C    
446	A A A A A A A A A A A A A A A A A A A A A A A A A A A A A A A A A A A A A A A A . A A A A A A A A A A A A A A A A A A A A A A A A A A A A . A A A A A    
447	A A A A A A A A A A A A A A A A A A A A A A A A A A A A A A A A A A A A A A A A . A A A A A A A A A A A A A A A A A A A A A A A A A A A A . A A A A A    
448	G G G G G G G G G G G G G G G G G G G G G G G G G G G G G G G G G G G G G G G G . G G G G G G G G G G G G G G G G G G G G G G G G G G G G . G G G G G    
449	A A A A A A A A A A A A A A A A A A A A A A A A A A A A A A A A A A A A A A A A . A A A A A A A A A A A A A A A A A A A A A A A A A A A A . A A A A A    
450	G G G G G G G G G G G G G G G G G G G G G G G G G G G G G G G G G G G G G G G G . G G G G G G G G G G G G G G G G G G G G G G G G G G G G . G G G G G    
451	A C C C C C C C C C C C C C C C C C C C C C C C C C C C C C C C C C C C C C C C . C C C C C C C C C C C C C C C C C C C C C C C C C C C C . C C C C C    
452	. C C C C C C C C C C C C C C C C C C C C C C C C C C C C C C C C C C C C C C C . C C C C C C C C C C C C C C C C C C C C C C C C C C C C . C C C C C    
453	. C C C C C C C C C C C C C C C C C C C C C C C C C C C C C C C C C C C C C C C . C C C C C C C C C C C C C C C C C C C C C C C C C C C C . C C C C C    
454	. T T T T T T T T T T T T T T T T T T T T T T T T T T T T T T T T T T T T T T T . T T T T T T T T T T T T T T T T T T T T T T T T T T T T . T T T T T    
455	. A A A A A A A A A A A A A A A A A A A A A A A A A A A A A A A A A A A A A A A . A A A A A A A A A A A A A A A A A A A A A A A A A A A A . A A A A A    
456	. G G G G G G G G G G G G G G G G G G G G G G G G G G G G G G G G G G G G G G G . G G G G G G G G G G G G G G G G G G G G G G G G G G G G . G G G G G    
457	. A A A A A A A A A A A A A A A A A A A A A A A A A A A A A A A A A A A A A A A . A A A A A A A A A A A A A A A A A A A A A A A A A A A A . A A A A A    
458	. A A A A A A A A A A A A A A A A A A A A A A A A A A A A A A A A A A A A A A A . A A A A A A A A A A A A A A A A A A A A A A A A A A A A . A A A A A    
459	. G G G G G G G G G G G G G G G G G G A G G G G G G G G G G G G G G G G G G G G . G G G G G G G G G G G G G G G G G G G G G G G G G G A G . G G G G G    
460	. C C C C C C C C C C C C C C C C C C C C C C C C C C C C C C C C C C C C C C C . C C C C C C C C C C C C C C C C C C C C C C C C C C C C . C C C C C    
461	. A A A A A A A A A A A A A A A A A A A A A A A A A A A A A A A A A A A A A A A . A A A A A A A A A A A A A A A A A A A A A A A A A A A A . A A A A A    
462	. A A A A A A A A A A A A A A A A A A A A A A A A A A A A A A A A A A A A A A A . A A A A A A A A A A A A A A A A A A A A A A A A A A A A . A A A A A    
463	. A A A A A A A A A A A A A A A A A A A A A A A A A A A A A A A A A A A A A A A . A A A A A A A A A A A A A A A A A A A A A A A A A A A A . A A A A A    
464	. C C C C C C C C C C C C C C C C C C C C C C C C C C C C C C C C C C C C C C C . C C C C C C C C C C C C C C C C C C C C C C C C C C C C . C C C C C    
465	. A A A A A A A A A A A A A A A A A A A A A A A A A A A A A A A A A A A A A A A . A A A A A A A A A A A A A A A A A A A A A A A A A A A A . A A A A A    
466	. - - - - - - - - - T - - - - - - T T - - - - - - - T - - - - - - - - - - - - - . - - T - - - - - - - - - - - T T T - - - - - - - - - - - . - - - - -  8	 indel  
467	. - - - - - - - - - T - - - - - - T T - - - - - - - T - - - - - - - - - - - - - . - - T - - - - - - - - - - - T T T - - - - - - - - - - - . - - - - -  8	 indel  
468	. - - - - - - - - - A - - - - - - A A - - - - - - - A - - - - - - - - - - - - - . - - A - - - - - - - - - - - A A A - - - - - - - - - - - . - - - - -  8	 indel  
469	. T T T T T T T T T T T T T T T T T T T T T T T T T T T T T T T T T T T T T T T . T T T T T T T T T T T T T T T T T T T T T T T T T T T T . T T T T T    
470	. T T T T T T T T T T T T T T T T T T T T T T T T T T T T T T T T T T T T T T T . T T T T T T T T T T T T T T T T T T T T T T T T T T T T . T T T T T    
471	. G G G G G G G G G G G G G G G G G G G G G G G G G G G G G G G G G G G G G G G . G G G G G G G G G G G G G G G G G G G G G G G G G G G G . G G G G G    
472	. G G G G G G G G G G G G G G G G G G G G G G G G G G G G G G G G G G G G G G G . G G G G G G G G G G G G G G G G G G G G G G G G G G G G . G G G G G    
473	. C C C C C C C C C C C C C C C C C C C C C C C C C C C C C C C C C C C C C C C . C C C C C C C C C C C C C C C C C C C C C C C C C C C C . C C C C C    
474	. T T T T T T T T T T T T T T T T T T T T T T T T T T T T T T T T T T T T T T T . T T T T T T T T T T T T T T T T T T T T T T T T T T T T . T T T T T    
475	. T T T T T T T T T T T T T T T T T T T T T T T T T T T T T T T T T T T T T T T . T T T T T T T T T T T T T T T T T T T T T T T T T T T T . T T T T T    
476	. A A A A A A A A A A A A A A A A A A A A A A A A A A A A A A A A A A A A A A A . A A A A A A A A A A A A A A A A A A A A A A A A A A A A . A A A A A    
477	. C C C C C C C C C C C C C C C C C C C C C C C C C C C C C C C C C C C C C C C . C C C C C C C C C C C C C C C C C C C C C C C C C C C C . C C C C C    
478	. A A A A A A A A A A A A A A A A A A A A A A A A A A A A A A A A A A A A A A A . A A A A A A A A A A A A A A A A A A A A A A A A A A A A . A A A A A    
479	. A A A A A A A A A A A A A A A A A A A A A A A A A A A A A A A A A A A A A A A . A A A A A A A A A A A A A A A A A A A A A A A A A A A A . A A A A A    
480	. C C C C C C C C C C C C C C C C C C C C C C C C C C C C C C C C C C C C C C C . C C C C C C C C C C C C C C C C C C C C C C C C C C C C . C C C C C    
481	. A A A A A A A A A A A A A A A A A A A A A A A A A A A A A A A A A A A A A A A . A A A A A A A A A A A A A A A A A A A A A A A A A A A A . A A A A A    
482	. A A A A A A A A A A A A A A A A A A A A A A A A A A A A A A A A A A A A A A A . A A A A A A A A A A A A A A A A A A A A A A A A A A A A . A A A A A    
483	. G G G G G G G G G G G G G G G G G G G G G G G G G G G G G G G G G G G G G G G . G G G G G G G G G G G G G G G G G G G G G G G G G G G G . G G G G G    
484	. A A A A A A A A A A A A A A A A A A A A A A A A A A A A A A A A A A A A A A A . A A A A A A A A A A A A A A A A A A A A A A A A A A A A . A A A A A    
485	. G G G G G G G G G G G G G G G G G G G G G G G G G G G G G G G G G G G G G G G . G G G G G G G G G G G G G G G G G G G G G G G G G G G G . G G G G G    
486	. G G G G G G G G G G G G G G G G G G G G G G G G G G G G G G G G G G G G G G G . G G G G G G G G G G G G G G G G G G G G G G G G G G G G . G G G G G    
487	. T T T T G T T T T T T T T T T G T T T T T T T T T T T T T T T T T T T T T T T . T T T T T T T T T T T T T T T T T T T T T T T T T T T T . T T T T T    
488	. G G G G G G G G G G G G G G G G G G G G G G G G G G G G G G G G G G G G G G G . G G G G G G G G G G G G G G G G G G G G G G G G G G G G . G G G G G    
489	. C C C C C C C C C C C C C C C C C C T C C C C C C C C C C C C C C C C C C C C . C C C C C C C C C C C C C C C C C C C C C C C C C C T C . C C C C C    
490	. A A A A A A A A A A A A A A A A A A A A A A A A A A A A A A A A A A A A A A A . A A A A A A A A A A A A A A A A A A A A A A A A A A A A . A A A A A    
491	. T T T T T T T T T T T T T T T T T T T T T T T T T T T T T T T T T T T T T T T . T T T T T T T T T T T T T T T T T T T T T T T T T T T T . T T T T T    
492	. T T T T T T T T T T T T T T T T T T T T T T T T T T T T T T T T T T T T T T T . T T T T T T T T T T T T T T T T T T T T T T T T T T T T . T T T T T    
493	. T T T T T T T T T T T T T T T T T T T T T T T T T T T T T T T T T T T T T T T . T T T T T T T T T T T T T T T T T T T T T T T T T T T T T T T T T T    
494	. G G G G G G G G G G G G G G G G G G G G G G G G G G G G G G G G G G G G G G G . G G G G G G G G G G G G G G G G G G G G G G G G G G G G G G G G G G    
495	. T T T T T T T T T T T T T T T T T T T T T T T T T T T T T T T T T T T T T T T . T T T T T T T T T T T T T T T T T T T T T T T T T T T T T T T T T T    
496	. A A A A A A A A A A A A A A A A A A A A A A A A A A A A A A A A A A A A A A A . A A A A A A A A A A A A A A A A A A A A A A A A A A A A A A A A A A    
497	. C C C C C C C C C C C C C C C C C C C C C C C C C C C C C C C C C C C C C C C . C C C C C C C C C C C C C C C C C C C C C C C C C C C C C C C C C C    
498	. A A A A A A A A A A A A A A A A A A A A A A A A A A A A A A A A A A A A A A A . A A A A A A A A A A A A A A A A A A A A A A A A A A A A A A A A A A    
499	. G G G G G G G G G G G G G G G G G G G G G G G G G G G G G G G G G G G G G G G . G G G G G G G G G G G G G G G G G G G G G G G G G G G G G G G G G G    
500	. A A A A A A A A A A A A A A A A A A A A A A A A A A A A A A A A A A A A A A A . A A A A A A A A A A A A A A A A A A A A A A A A A A A A A A A A A A    
501	. T T T T T T T T T T T T T T T T T T T T T T T T T T T T T T T T T T T T T T T . T T T T T T T T T T T T T T T T T C T T T T T T T T T T T T T T T T    
502	. G G G G G G G G G G G G G G G G G G G G G G G G G G G G G G G G G G G G G G G . G G G G G G G G G G G G G G G G G G G G G G G G G G G G G G G G G G    
503	. T T T T T T T T T T T T T T T T T T T T T T T T T T T T T T T T T T T T T T T . T T T T T T T T T T T T T T T T T T T T T T T T T T T T T T T T T T    
504	. C C C C C C C C C C C C C C C C C C C C C C C C C C C C C C C C C C C C C C C . C C C C C C C C C C C C C C C C C C C C C C C C C C C C C C C C C C    
505	. A A A A A A A A A A A A A A A A A A A A A A A A A A A A A A A A A A A A A A A . A A A A A A A A A A A A A A A A A A A A A A A A A A A A A A A A A A    
506	. A A A A A A A A A A A A A A A A A A A A A A A A A A A A A A A A A A A A A A A . A A A A A A A A A A A A A A A A A A A A A A A A A A A A A A A A A A    
507	. A A A A A A A A A A A A A A A A A A A A A A A A A A A A A A A A A A A A A A A . A A A A A A A A A A A A A A A A A A A A A A A A A A A A A A A A A A    
508	. A A A A A A A A A A A A A A A A A A A A A A A A A A A A A A A A A A A A A A A . A A A A A A A A A A A A A A A A A A A A A A A A A A A A A A A A A A    
509	. T T T T T T T T T T T T T T T T T T T T T T T T T T T T T T T T T T T T T T T . T T T T T T T T T T T T T T T T T T T T T T T T T T T T T T T T T T    
510	. G G G G G G G G G G G G G G G G G G G G G G G G G G G A G G G G G G G G G G G . G G G G G G G G G G G G G G G G G G G G G G G A G G G G G G G G G G    
511	. C C C C C C C C C C C C C C C C C C C C C C C C C C C C C C C C C C C C C C C . C C C C C C C C C C C C C C C C C C C C C C C C C C C C C C C C C C    
512	. A A A A A A A A A A A A A A A A A A A A A A A A A A A A A A A A A A A A A A A . A A A A A A A A A A A A A A A A A A A A A A A A A A A A A A A A A A    
513	. C C C C C C C C C C C C C C C C C C C C C C C C C C C C C C C C C C C C C C C . C C C C C C C C C C C C C C C C C C C C C C C C C C C C C C C C C C    
514	. A A A A A A A A A A A A A A A A A A A A A A A A A A A A A A A A A A A A A A A . A A A A A A A A A A A A A A A A A A A A A A A A A A A A A A A A A A    
515	. T T T T T T T T T T T T T T T T T T T T T T T T T T T T T T T T T T T T T T T . T T T T T T T T T T T T T T T T T T T T T T T T T T T T T T T T T T    
516	. T T T T T T T T T T T T T T T T T T T T T T T T T T T T T T T T T T T T T T T . T T T T T T T T T T T T T T T T T T T T T T T T T T T T T T T T T T    
517	. C C C C C C C C C C C C C C C C C C C C C C C C C C C C C C C C C C C C C C C . C C C C C C C C C C C C C C C C C C C C C C C C C C C C C C C C C C    
518	. T T T T T T T T T T T T T T T T T T T T T T T T T T T T T T T T T T T T T T T . T T T T T T T T T T T T T T T T T T T T T T T T T T T T T T T T T T    
519	. G G G G G G G G G G G G G G G G G G G G G G G G G G G G G G G G G G G G G G G . G G G G G G G G G G G G G G G G G G G G G G G G G G G G G G G G G G    
520	. T T T T T T T T T T T T T T T T T T T T T T T T T T T T T T T T T T T T T T T . T T T T T T T T T T T T T T T T T T T T T T T T T T T T T T T T T T    
521	. G G G G G G G G G G G G G G G G G G G G G G G G G G G G G G G G G G G G G G G . G G G G G G G G G G G G G G G G G G G G G G G G G G G G G G G G G G    
522	. A A A A A A A A A A A A A A A A A A A A A A A A A A A A A A A A A A A A A A A . A A A A A A A A A A A A A A A A A A A A A A A A A A A A A A A A A A    
523	. C C C C C C C C C C C C C C C C C C C C C C C C C C C C C C C C C C C C C C C . C C C C C C C C C C C C C C C C C C C C C C C C C C C C C C C C C C    
524	. A A A A A A A A A A A A A A A A A A A A A A A A A A A A A A A A A A A A A A A . A A A A A A A A A A A A A A A A A A A A A A A A A A A A A A A A A A    
525	. A A A A A A A A A A A A A A A A A A A A A A A A A A A A A A A A A A A A A A A . A A A A A A A A A A A A A A A A A A A A A A A A A A A A A A A A A A    
526	. A A G A A A A A A A A A A A A A A A A A A A A A A A A A G A A A A A A A A A A . A A A A A A A A A A A A A A A A A A A A A A A A A A A A A A A A A A    
527	. G G G G G G G G G G G G G G G G G G G G G G G G G G G G G G G G G G G G G G G . G G G G G G G G G G G G G G G G G G G G G G G G G G G G G G G G G G    
528	. G G G G G G G G G G G G G G G G G G G G G G G G G G G G G G G G G G G G G G G . G G G G G G G G G G G G G G G G G G G G G G G G G G G G G G G G G G    
529	. G G G G G G G G G G G G G G G G G G G G G G G G G G G G G G G G G G G G G G G . G G G G G G G G G G G G G G G G G G G G G G G G G G G G G G G G G G    
530	. G G G G G G G G G G G G G G G G G G G G G G G G G G G G G G G G G G G G G G G . G G G G G G G G G G G G G G G G G G G G G G G G G G G G G G G G G G    
531	. G G G G G G G G G G G G G G G G G G G G G G G G G G G G G G G G G G G G G G G . G G G G G G G G G G G G G G G G G G G G G G G G G G G G G G G G G G    
532	. A A A A A A A G A A A A A A A A A A A A A A A A A A A A A A A A A A A A A A A . A A A A A A A A A A G A A A A A A A A A A A A A A A A A A A A A A A    
533	. A A A A A A A A A A A A A A A A A A A A A A A A A A A A A A A A A A A A A A A . A A A A A A A A A A A A A A A A A A A A A A A A A A A A A A A A A A    
534	. G G G G G G G G G A G G G G G G G G G G G G G G G G G G G G G G G G G G G G G . G G G G G G G G G G G G G G G G G G G G G G G G G G G G G G G G G G    
535	. A A A A A A A A A A A A A A A A A A A A A A A A A A A A A A A A A A A A A A A . A A A A A A A A A A A A A A A A A A A A A A A A A A A A A A A A A A    
536	. G G G G G G G G G G G G G G G G G G G G G G G G G G G G G G G G G G G G G G G . G G G G G G G G G G G G G G G G G G G G G G G G G G G G G G G G G G    
537	. G G G G G G G G G G G G G G G G G G G G G G G G G G G G G G G G G G G G G G G . G G G G G G G G G G G G G G G G G G G G G G G G G G G G G G G G G G    
538	. G G G G G G G G G G G G G G G G G G G G G G G G G G G G G G G G G G G G G G G . G G G G G G G G G G G G G G G G G G G G G G G G G G G G G G G G G G    
539	. G G G G G G G G G G G G G G G G G G G G G G G G G G G G G G G G G G G G G G G . G G G G G G G G G G G G G G G G G G G G G G G G G G G G G G G G G G    
540	. A A A A A A A A A A A A A A A A A A A A A A A A A A A A A A A A A A A A A A A . A A A A A A A A A A A A A A A A A A A A A A A A A A A A A A A A A A    
541	. G G G G G G G G G G G G G G G G G G G G G G G G G G G G G G G G G G G G G G G . G G G G G G G G G G G G G G G G G G G G G G G G G G G G G G G G G G    
542	. A A A A A A A A A A A A A A A A A A A A A A A A A A A A A A A A A A A A A A A . A A A A A A A A A A A A A A A A A A A A A A A A A A A A A A A A A A    
543	. C C C C C C C C C C C C C C C C C C C C C C C C C C C C C C C C C C C C C C C . C C C C C C C C C C C C C C C C C C C C C C C C C C C C C C C C C C    
544	. T T T T T T T T T T T T T T T T T T T T T T T T T T T T T T T T T T T T T T T . T T T T T T T T T T T T T T T T T T T T T T T T T T T T T T T T T T    
545	. A A A A A A A A A A A A A A A A A A A A A A A A A A A A A A A A A A A A A A A . A A A A A A A A A A A A A A A A A A A A A A A A A A A A A A A A A A    
546	. T T T T T T T T T T T T T T T T T T T T T T T T T T T T T T T T T T T T T T T . T T T T T T T T T T T T T T T T T T T T T T T T T T T T T T T T T T    
547	. T T T T T T T T T T T T T T T T T T T T T T T T T T T T T T T T T T T T T T T . T T T T T T T T T T T T T T T T T T T T T T T T T T T T T T T T T T    
548	. T T T T T T T T T T T T T T T T T T T T T T T T T T T T T T T T T T T T T T T . T T T T T T T T T T T T T T T T T T T T T T T T T T T T T T T T T T    
549	. T T T T T T T T T T T T T T T T T T T T T T T T T T T T T T T T T T T T T T T . T T T T T T T T T T T T T T T T T T T T T T T T T T T T T T T T T T    
550	. G G G G G G G G G G G G G G G G G G G G G G G G G G G G G G G G G G G G G G G . G G G G G G G G G G G G G G G G G G G G G G G G G G G G G G G G G G    
551	. G G G G G G G G G G G G G G G G G G G G G G G G G G G G G G G G G G G G G G G . G G G G G G G G G G G G G G G G G G G G G G G G G G G G G G G G G G    
552	. G G G G G G G G G G G G G G G G G G G G G G G G G G G G G G G G G G G G G G G . G G G G G G G G G G G G G G G G G G G G G G G G G G G G G G G G G G    
553	. G G G G G G G G G G G G G G G G G G G G G G G G G G G G G G G G G G G G G G G . G G G G G G G G G G G G G G G G G G G G G G G G G G G G G G G G G G    
554	. G G G G G G G G G G G G G G G G G G G G G G G G G G G G G G G G G G G G G G G . G G G G G G G G G G G G G G G G G G G G G G G G G G G G G G G G G G    
555	. A A A A A A A A A A A A A A A A A A A A A A A A A A A A A A A A A A A A A A A . A A A A A A A A A A A A A A A A A A A A A A A A A A A A A A A A A A    
556	. T T T T T T T T T T T T T T T T T T T T T T T T T T T T T T T T T T T T T T T . T T T T T T T T T T T T T T T T T T T T T T T T T T T T T T T T T T    
557	. C C . C C C C C C C C C C C C C C C C C C C C C C C C C C C C C C C C C C C C . C C C C C C C C C C C C C C C C C C C C C C C C C C C C C C C C C C    
558	. T T . T T T T T T T T T T T T T T T T T T T T T T T T T T T T T T T T T T T T . T T T T T T T T T T T T T T T T T T T T T T T T T T T T T T T T T T    
559	. . G . G G G G G G G G G G G G G G G G G G G G G G G G G G G G G G G G G G G G . G G G G G G G G G G G G G G G G G G G G G G G G G G G G G G G G G G    
560	. . T . T G T T T T T T T T T T G T T T T T T T T T T T T T T T T T T T T T T T . T T T T T T T T T T T T T T T T T T T T T T T T T T T T T T T T T T    
561	. . G . G G G G G G A G G G G G G G G G G G G G G G G G G G G G G G G G G G G G . G G G G G G G G G G G G G G A G G G G G G G G G G G G G G G G G G G    
562	. . T . T T T T T T T T T T T T T T T T T T T T T T T T T T T T T T T T T T T T . T T T T T T T T T T T T T T T T T T T T T T T T T T T T T T T T T T    
563	. . T . T T T T T T T T T T T T T T T T T T T T T T T T T T T T T T T T T T T T . T T T T T T T T T T T T T T T T T T T T T T T T T T T T T T T T T T    
564	. . G . G G G G G G G G G G G G G G G G G G G G G G G G G G G G G G G G G G G G . G G G G G G G G G G G G G G G G G G G G G G G G G G G G G G G G G G    
565	. . A . A A A A A A A A A A A A A A A A A A A A A A A A A A A A A A A A A A A A . A A A A A A A A A A A A A A A A A A A A A A A A A A A A A A A A A A    
566	. . T . T T T T T T T T T T T T T T T T T T T T T T T T T T T T T T T T T T T T . T T T T T T T T T T T T T T T T T T T T T T T T T T T T T T T T T T    
567	. . G . G G G G G G G G G G G G G G G G G G G G G G G G G G G G G G G G G G G G . G G G G G G G G G G G G G G G G G G G G G G G G G G G G G G G G G G    
568	. . C . C C C C C C C C C C C C C C C C C C C C C C C C C C C C C C C C C C C C . C C C C C C C C C C C C C C C C C C C C C C C C C C C C C C C C C C    
569	. . C . C C C C C C C C C C C C C C C C C C C C C C C C C C C C C C C C C C C C . C C C C C C C C C C C C C C C C C C C C C C C C C C C C C C C C C C    
570	. . A . A A A A A A A A A A A A A A A A A A A A A A A A A A A A A A A A A A A A . A A A A A A A A A A A A A A A A A A A A A A A A A A A A A A A A A A    
571	. . T . T T T T T T T T T T T T T T T T T T T T T T T T T T T T T T T T T T T T . T T T T T T T T T T T T T T T T T T T T T T T T T T T T T T T T T T    
572	. . A . A A A A A A A A A A A A A A A A A A A A A A A A A A A A A A A A A A A A . A A A A A A A A A A A A A A A A A A A A A A A A A A A A A A A A A A    
573	. . T . T T T T T T T T T T T T T T T T T T T T T T T T T T T T T T T T T T T T . T T T T T T T T T T T T T T T T T T T T T T T T T T T T T T T T T T    
574	. . T . T T T T T T T T T T T T T T T T T T T T T T T T T T T T T T T T T T T T . T T T T T T T T T T T T T T T T T T T T T T T T T T T T T T T T T T    
575	. . T . T T T T T T T T T T T T T T T T T T T T T T T T T T T T T T T T T T T T . T T T T T T T T T T T T T T T T T T T T T T T T T T T T T T T T T T    
576	. . C . C C C C C C C C C C C C C C C C C C C C C C C C C C C C C C C C C C C C . C C C C C C C C C C C C C C C C C C C C C C C C C C C C C C C C C C    
577	. . C . T T T T T T T T T T T T T T T T T T T T T T T T T T T T T T T T T T T T . T T T T T T T T T T T T T T T T T T T T T T T T T T T T T T T T T T    
578	. . . . C C C C C C C C C C C C C C C C C C C C C C C C C C C C C C C C C C C C . C C C C C C C C C C C C C C C C C C C C C C C C C C C C C C C C C C    
579	. . . . C C C C C C C C C C C C C C C C C C C C C C C C C C C C C C C C C C C C . C C C C C C C C C C C C C C C C C C C C C C C C C C C C C C C C C C    
580	. . . . T T T T T T T T T T T T T T T T T T T T T T T T T T T T T T T T T T T T . T T T T T T T T T T T T T T T T T T T T T T T T T T T T T T T T T T    
581	. . . . T T T T T T T T T T T T T T T T T T T T T T T T T T T T T T T T T T T T . T T T T T T T T T T T T T T T T T T T T T T T T T T T T T T T T T T    
582	. . . . C C C C C C C C C C C C C C C C C C C C C C C C C C C C C C C C C C C C . C C C C C C C C C C C C C C C C C C C C C C C C C C C C C C C C C C    
583	. . . . A A A A A A A A A A A A A A A A A A A A A A A A A A A A A A A A A A A A . A A A A A A A A A A A A A A A A A A A A A A A A A A A A A A A A A A    
584	. . . . G G G G G G G G G G G G G G G G G G G G G G G G G G G G G G G G G G G G . G G G G G G G G G G G G G G G G G G G G G G G G G G G G G G G G G G    
585	. . . . A A A A A A A A A A A A A A A A A A A A A A A A A A A A A A A A A A A A . A A A A A A A A A A A A A A A A A A A A A A A A A A A A A A A A A A    
586	. . . . A A A A A A A A A A A A A A A A A A A A A A A A A A A A A A A A A A A A . A A A A A A A A A A A A A A A A A A A A A A A A A A A A A A A A A A    
587	. . . . G G G G G G G G G G G G G G G G G G G G G G G G G G G G G G G G G G G G . G G G G G G G G G G G G G G G G G G G G G G G G G G G G G G G G G G    
588	. . . . C C C C C C C C C C C C C C C C C C C C C C C C C C C C C C C C C C C C . C C C C C C C C C C C C C C C C C C C C C C C C C C C C C C C C C C    
589	. . . . T T T T T T T T T T T T T T T T T T T T T T T T T T T T T T T T T T T T . T T T T T T T T T T T T T T T T T T T T T T T T T T T T T T T T T T    
590	. . . . T T T T T T T T T T T T T T T T T T T T T T T T T T T T T T T T T T T T . T T T T T T T T T T T T T T T T T T T T T T T T T T T T T T T T T T    
591	. . . . C C C C C C C C C C C C C C C C C C C C C C C C C C C C C C C C C C C C . C C C C C C C C C C C C C C C C C C C C C C C C C C C C C C C C C C    
592	. . . . A A A A A A A A A A A A A A A A A A A A A A A A A A A A A A A A A A A A . A A A A A A A A A A A A A A A A A A A A A A A A A A A A A A A A A A    
593	. . . . G G G G G G G G G G G G G G G G G G G G G G G G G G G G G G G G G G G G . G G G G G G G G G G G G G G G G G G G G G G G G G G G G G G G G G G    
594	. . . . C C C C C C C C C C C C C C C C C C C C C C C C C C C C C C C C C C C C . C C C C C C C C C C C C C C C C C C C C C C C C C C C C C C C C C C    
595	. . . . T T T T T T T T T T T T T T T T T T T T T T T T T T T T T T T T T T T T . T T T T T T T T T T T T T T T T T T T T T T T T T T T T T T T T T T    
596	. . . . G G G G G G G G G G G G G G G G G G G G G G G G G G G G G G G G G G G G . G G G G G G G G G G G G G G G G G G G G G G G G G G G G G G G G G G    
597	. . . . C C C C C C C C C C C C C C C C C C C C C C C C C C C C C C C C C C C C . C C C C C C C C C C C C C C C C C C C C C C C C C C C C C C C C C C    
598	. . . . T T T T T T T T T T T T T T T T T T T T T T T T T T T T T T T T T T T T . T T T T T T T T T T T T T T T T T T T T T T T T T T T T T T T T T T    
599	. . . . C C C C C C C C C C C C C C C C C C C C C C C C C C C C C C C C C C C C . C C C C C C C C C C C C C C C C C C C C C C C C C C C C C C C C C C    
600	. . . . C C C C C C C C C C C C C C C C C C C C C C C C C C C C C C C C C C C C . C C C C C C C C C C C C C C C C C C C C C C C C C C C C C C C C C C    
601	. . . . T T T T T T T T T T T T T T T T T T T T T T T T T T T T T T T T T T T T . T T T T T T T T T T T T T T T T T T T T T T T T T T T T T T T T T T    
602	. . . . C C C C C C C C C C C C C C C C C C C C C C C C C C C C C C C C C C C C . C C C C C C C C C C C C C C C C C C C C C C C C C C C C C C C C C C    
603	. . . . T T T T T T T T T T T T T T T T T T T T T T T T T T T T T T T T T T T T . T T T T T T T T T T T T T T T T T T T T T T T T T T T T T T T T T T    
604	. . . . T T T T T T T T T T T T T T T T T T T T T T T T T T T T T T T T T T T T . T T T T T T T T T T T T T T T T T T T T T T T T T T T T T T T T T T    
605	. . . . T T T T T T T T T T T T T T T T T T T T T T T T T T T T T T T T T T T T . T T T T T T T T T T T T T T T T T T T T T T T T T T T T T T T T T T    
606	. . . . C C C C C C C C C C C C C C C C C C C C C C C C C C C C C C C C C C C C . C C C C C C C C C C C C C C C C C C C C C C C C C C C C C C C C C C    
607	. . . . T C T T T T T T T T T T T T T T T T T T T T T T T T T T T T T T T T T T . T T T T T T T T T T T T T T T T T T T T T T T T T T T T T T T T T T    
608	. . . . T . T T T T T T T T T T T T T T T T T T T T T T T T T T T T T T T T T T . T T T T T T T T T T T T T T T T T T T T T T T T T T T T T T T T T T    
609	. . . . C . C C - C C C C C C C C C C C C C C C C C C C C C C C C C C C C C C C . C C C C C C C C C C - C C C C C C C C C C C C C C C C C C C C C C C    
610	. . . . T . T T - T T T T T T T T T T T T T T T T T T T T T T T T T T T T T T T . T T T T T T T T T T - T T T T T T T T T T T T T T T T T T T T T T T    
611	. . . . T . T T - T T T T T T T T T T T T T T T T T T T T T T T T T T T T T T T . T T T T T T T T T T - T T T T T T T T T T T T T T T T T T T T T T T    
612	. . . . G . G G G G G G G G G G G G G G G G G G G G G G G G G G G G G G G G G G . G G G G G G G G G G G G G G G G G G G G G G G G G G G G G G G G G G    
613	. . . . . . G . G G G G G G G G G G G G G G G G G G G G G G G G G G G G G G G G . G G A G G G G G G G G G G G G G G G G G G G G G G G G G G G G G G G    
614	. . . . . . T . T T T G T T T T T T T T T T T T T T T T T T T T T T T T T T T T . T T T T T T T T T T T T T T T T T T T T T T T T T T T T T T T T T T    
615	. . . . . . G . G G G G G G G G G G G G G G G G G G G G G G G G G G G G G G G G . G G G G G G G G G G G G G G G G G G G G G G G G G G G G G G G G G G    
616	. . . . . . T . T T T T T T T T T T T T T T T T T T T T T T T T T T T T T T T T . T T T T T T T T T T T T T T T T T T T T T T T T T T T T T T T T T T    
617	. . . . . . C . C C C C C C C C C C C C C C C C C C C C C C C C C C C C C C C C . C C C C C C C C C C C C C C C C C C C C C C C C C C C C C C C C C C    
618	. . . . . . C . C C C C C C C C C C C C C C C C C C C C C C C C C C C C C C C C . C C C C C C C C C C C C C C C C C C C C C C C C C C C C C C C C C C    
619	. . . . . . A . A A A A A A A A A A A A A A A A A A A A A A A A A A A A A A A A . A A A A A A A A A A A A A A A A A A A A A A A A A A A A A A A A A A    
620	. . . . . . T . T T T T T T T T T T T T T T T T T T T T T T T T T T T T T T T T . T T T T T T T T T T T T T T T T T T T T T T T T T T T T T T T T T T    
621	. . . . . . C . C C C C C C C C C C C C C C C C C C C C C C C C C C C C C C C C . C C C C C C C C C C C C C C C C C C C C C C C C C C C C C C C C C C    
622	. . . . . . T . T T T T T T T T T T T T T T T T T T T T T T T T T T T T T T T T . T T T T T T T T T T T T T T T T T T T T T T T T T T T T T T T T T T    
623	. . . . . . T . T T T T T T T T T T T T T T T T T T T T T T T T T T T T T T T T . T T T T T T T T T T T T T T T T T T T T T T T T T T T T T T T T T T    
624	. . . . . . C . C C C C C C C C C C C C C C C C C C C C C C C C C C C C C C C C . C C C C C C C C C C C C C C C C C C C C C C C C C C C C C C C C C C    
625	. . . . . . T . T T T T T T T T T T T T T T T T T T T T T T T T T T T T T T T T . T T T T T T T T T T T T T T T T T T T T T T T T T T T T T T T T T T    
626	. . . . . . G . G G G G G G G G G G G G G G G G G G G G G G G G G G G G G G G G . G G G G G G G G G G G G G G G G G G G G G G G G G G G G G G G G G G    
627	. . . . . . G . G G G G G G G G G G G G G G G G G G G G G G G G G G G G G G G G . G G G G G G G G G G G G G G G G G G G G G G G G G G G G G G G G G G    
628	. . . . . . A . A A A A A A A A A A A A A A A A A A A A A A A A A A A A A A A A . A A A A A A A A A A A A A A A A A A A A A A A A A A A A A A A A A A    
629	. . . . . . A . A A A A A A A A A A A A A A A A A A A A A A A A A A A A A A A A . A A A A A A A A A A A A A A A A A A A A A A A A A A A A A A A A A A    
630	. . . . . . A . A A A A A A A A A A A A A A A A A A A A A A A A A A A A A A A A . A A A A A A A A A A A A A A A A A A A A A A A A A A A A A A A A A A    
631	. . . . . . G . G G G G G G G G G G G G G G G G G G G G G G G G G G G G G G G G . G G G G G G G G G G G G G G G G G G G G G G G G G G G G G G G G G G    
632	. . . . . . C . C C C C C C C C C C C C C C C C C C C C C C C C C C C C C C C C . C C C C C C C C C C C C C C C C C C C C C C C C C C C C C C C C C C    
633	. . . . . . C . C C C C C C C C C C C C C C C C C C C C C C C C C C C C C C C C . C C C C C C C C C C C C C C C C C C C C C C C C C C C C C C C C C C    
634	. . . . . . C . C C C C C C T C C C C C C C C C C C C C C C C C C C C C C C C C . C C C C C C C C C C C C C C C C C C C C C C C C C C C C C C C C C C    
635	. . . . . . T . T T T T T T T T T T T T T T T T T T T T T T T T T T T T T T T T . T T T T T T T T T T T T T T T T T T T T T T T T T T T T T T T T T T    
636	. . . . . . G . G G G G G G G G G G G G G G G G G G G G G G G G G G G G G G G G . G G G G G G G G G G G G G G G G G G G G G G G G G G G G G G G G G G    
637	. . . . . . T . T T T T T T T T T T T T T T T T T T T T T T T T T T T T T T T T . T T T T T T T T T T T T T T T T T T T T T T T T T T T T T T T T T T    
638	. . . . . . C . T T T T T T T T T T T T T T T T T T T T T T T T T T T T T T T T . T T T T T T T T T T T T T T T T T T T T T T T T T T T T T T T T T T    
639	. . . . . . . . T T C T T T T T T T T T T T T T T T T T T T T T T T T T T T T T . T T T T T T T T T T T T T T C T T T T T T T T T T T T T T T T T T T    
640	. . . . . . . . G G G G G G G G G G G G G G G G G G G G G G G G G G G G G G G G . G G G G G G G G G G G G G G G G G G G G G G G G G G G G G G G G G G    
641	. . . . . . . . C C C C C C C C C C C C C C C C C C C C C C C C C C C C C C C C . C C C C C C C C C C C C C C C C C C C C C C C C C C C C C C C C C C    
642	. . . . . . . . C A A A A A A A A A A A A A A A A A A A A A A A A A A A A A A A A A A A A A A A A A A A A A A A A A A A A A A A A A A A A A A A A A A    
643	. . . . . . . . . T T T T T T T T T T T T T T T T T T T T T T T T T T T T T T T T T T T T T T T T T T T T T T T T T T T T T T T T T T T T T T T T T T    
644	. . . . . . . . . T T T T T T T T T T T T T T T T T T T T T T T T T T T T T T T T T T T T T T T T T T T T T T T T T T T T T T T T T T T T T T T T T T    
645	. . . . . . . . . G . G G G G G G G G G G G G G G G G G G G G G G G G G G G G G G G G G G G G G G G G G G G G G G G G G G G G G G G G G G G G G G G G    
646	. . . . . . . . . T . T T T T T T T T T T T T T T T T T T T T T T T T T T T T T T T T T T T T T T T T T T T T T T T T T T T T T T T T T T T T T T T T    
647	. . . . . . . . . A . A A A A A A A A A A A A A A A A A A A A A A A A A A A A A A A A A A A A A A A A A A A A A A A A A A A A A A A A A A A A A A A A    
648	. . . . . . . . . G . G G G G G G G G G G G G G G G G G G G G G G G G G G G G G G G G G G G G G G G G G G G G G G G G G G G G G G G G G G G G G G G G    
649	. . . . . . . . . T . T T T T T T T T T T T T T T T T T T T T T T T T T T T T T T T T T T T T T T T T T T T T T T T T T T T T T T T T T T T T T T T T    
650	. . . . . . . . . A . A A A A A A A A A A A A A A A A A A A A A A A A A A A A A A A A A A A A A A A A A A A A A A A A A A A A A A A A A A A A A A A A    
651	. . . . . . . . . T . T T T T T T T T T T T T T T T T T T T T T T T T T T T T T T T T T T T T T T T T T T T T T T T T T T T T T T T T T T T T T T T T    
652	. . . . . . . . . A . A A A A A A A A A A A A A A A A A A A A A A A A A A A A A A A A A A A A A A A A A A A A A A A A A A A A A A A A A A A A A A A A    
653	. . . . . . . . . A . A A A A A A A A A A A A A A A A A A A A A A A A A A A A A A A A A A A A A A A A A A A A A A A A A A A A A A A A A A A A A A A A    
654	. . . . . . . . . A . A A A A A A A A A A A A A A A A A A A A A A A A A A A A A A A A A A A A A A A A A A A A A A A A A A A A A A A A A A A A A A A A    
655	. . . . . . . . . A . A A A A A A A A A A A A A A A A A A A A A A A A A A A A A A A A A A A A A A A A A A A A A A A A A A A A A A A A A A A A A A A A    
656	. . . . . . . . . C . C C C C C C C C C C C C C C C C C C C C C C C C C C C C C C C C C C C C C C C C C C C C C C C C C C C C C C C C C C C C C C C C    
657	. . . . . . . . . T . T T T T T T T T T T T T T T T T T T T T T T T T T T T T T T T T T T T T T T T T T T T T T T T T T T T T T T T T T T T T T T T T    
658	. . . . . . . . . A . A A A A A A A A A A A A A A A A A A A A A A A A A A A A A A A A A A A A A A A A A A A A A A A A A A A A A A A A A A A A A A A A    
659	. . . . . . . . . C . C C C C C C C C C C C C C C C C C C C C C C C C C C C C C C C C C C C C C C C C C C C C C C C C C C C C C C C C C C C C C C C C    
660	. . . . . . . . . A . A A A A A A A A A A A A A A A A A A A A A A A A A A A A A A A A A A A A A A A A A A A A A A A A A A A A A A A A A A A A A A A A    
661	. . . . . . . . . . . A A A A A A A A A A A A A A A A A A A A A A A A A A A A A A A A A A A A A A A A A A A A A A A A A A A A A A A A A A A A A A A A    
662	. . . . . . . . . . . G G G G G G G G G G G G G G G G G G G G G G G G G G G G G G G G G G G G G G G G G G G G G G G G G G G G G G G G G G G G G G G G    
663	. . . . . . . . . . . A A A A A A A A A A A A A A A A A A A A A A A A A A A A A A A A A A A A A A A A A A A A A A A A A A A A A A A A A A A A A A A A    
664	. . . . . . . . . . . T T T T T T T T T T T T T T T T T T T T T T T T T T T T T T T T C T T T T T T T T T T T T T T T T T T T T T T T T T T T T T T T    
665	. . . . . . . . . . . A A A A A A A A A A A A A A A A A A A A A A A A A A A A A A A A A A A A A A A A A A A A A A A A A A A A A A A A A A A A A A A A    
666	. . . . . . . . . . . T T T T T T T T T T T T T T T T T T T T T T T T T T T T T T T T T T T T T T T T T T T T T T T T T T T T T T T T T T T T T T T T    
667	. . . . . . . . . . . C C C C C C C C C C C C C C C C C C C C C C C C C C C C C C C C C C C C C C C C C C C C C C C C C C C C C C C C C C C C C C C C    
668	. . . . . . . . . . . T T T T T T T T T T T T T T T T T T T T T T T T T T T T T T T T T T T T T T T T T T T T T T T T T T T T T T T T T T T T T T T T    
669	. . . . . . . . . . . G G G G G G G G G G G G G G G G G G G G G G G G G G G G G G G G G G G G G G G G G G G G G G G G G G G G G G G G G G G G G G G G    
670	. . . . . . . . . . . T T T T T T T T T T T T T T T T T T C T T T T T T T T T T T T T T T T T T T T T T T T T T T T T T T T T T T T T T T T T T T T T    
671	. . . . . . . . . . . . T T T T T T T T T T T T T T T T T T T T T T T T T T T T T T T T T T T T T T T T T T T T T T T T T T T T T T T T T T T T T T T    
672	. . . . . . . . . . . . G G G G G G G G G G G G G G G G G G G G G G G G G G G G G G G G G G G G G G G G G G G G G G G G G G G G G G G G G G G G G G G    
673	. . . . . . . . . . . . C C C C C C C C C C C C C C C C C C C C C C C C C C C C C C C C C C C C C C C C C C C C C C C C C C C C C C C C C C C C C C C    
674	. . . . . . . . . . . . A A A A A A A A A A A A A A A A A A A A A A A A A A A A A A A A A A A A A A A A A A A A A A A A A A A A A A A A A A A A A A A    
675	. . . . . . . . . . . . G G G G G G G G G G G G G G G G G G G G G G G G G G G G G G G G G G G G G G G G G G G G G G G G G G G G G G G G G G G G G G G    
676	. . . . . . . . . . . . A A A A A . A A A A A A A A A A A A A A A A A A A A A A A A A A A A A A A A A A A A A A A A A A A A A A A A A A A A A A A A A    
677	. . . . . . . . . . . . C C C C C . C C C C C C C C C C C C C C C C C C C C C C C C C C C C C C C C C C C C C C C C C C C C C C C C C C C C C C C C C    
678	. . . . . . . . . . . . G G G G G . G G G G G G G G G G G G G G G G G G G G G G G G G G G G G G G G G G G G G G G G G G G G G G G G G G G G G G G G G    
679	. . . . . . . . . . . . T T T T T . T T T T T T T T T T T T T T T T T T T T T T T T T T T T T T T T T T T T T T T T T T T T T T T T T T T T T T T T T    
680	. . . . . . . . . . . . T T T T T . T T T T T T T T T T T T T T T T T T T T T T T T T T T T T T T T T T T T T T T T T T T T T T T T T T T T T T T T T    
681	. . . . . . . . . . . . G G G G G . G G G G G G G G G G G G G G G G G G G G G G G G G G G G G G G G G G G G G G G G G G G G G G G G G G G G G G G G G    
682	. . . . . . . . . . . . A A A A A . A A A A A A A A A A A A A A A A A A A A A A A A A A A A A A A A A A A A A A A A A A A A A A A A A A A A A A A A A    
683	. . . . . . . . . . . . A A A A A . A A A A A A A A A A A A A A A A A A A A A A A A A A A A A A A A A A A A A A A A A A A A A A A A A A A A A A A A A    
684	. . . . . . . . . . . . T T T T T . T T T T T T T T T T T T T T T T T T T T T T T T T T T T T T T T T T T T T T T T T T T T T T T T T T T T T T T T T    
685	. . . . . . . . . . . . C C C C C . C C C C C C C C C C C C C C C C C C C C C C C C C C C C C C C C C C C C C C C C C C C C C C C C C C C C C C C C C    
686	. . . . . . . . . . . . C C C C C . C C C C C C C C C C C C C C C C C C C C C C C C C C C C C C C C C C C C C C C C C C C C C C C C C C C C C C C C C    
687	. . . . . . . . . . . . A A A A A . A A A A A A A A A A A A A A A A A A A A A A A A A A A A A A A A A A A A A A A A A A A A A A A A A A A A A A A A A    
688	. . . . . . . . . . . . G G G G G . G G G G G G G G G G G G G G G G G G G G G G G G G G G G G G G G G G G G G G G G G G G G G G G G G G G G G G G G G    
689	. . . . . . . . . . . . A A A A A . A A A A A A A A A A A A A A A A A A A A A A A A A A A A A A A A A A A A A A A A A A A A A A A A A A A A A A A A A    
690	. . . . . . . . . . . . A A A A A . A A A A A A A A A A A A A A A A A A A A A A A A A A A A A A A A A A A A A A A A A A A A A A A A A A A A A A A A A    
691	. . . . . . . . . . . . A A A A A . A A A A A A A A A A A A A A A A A A A A A A A A A A A A A A A A A A A A A A A A A A A A A A A A A A A A A A A A A    
692	. . . . . . . . . . . . G G G G G . G G G G G G G G G G G G G G G G G G G G G G G G G G G G G G G G G G G G G G G G G G G G G G G G G G G G G G G G G    
693	. . . . . . . . . . . . G G G G G . G G G G G G G G G G G G G G G G G G G G G G G G G G G G G G G G G G G G G G G G G G G G G G G G G G G G G G G G G    
694	. . . . . . . . . . . . T G T T T . T T T T T T T T T T T T T T T T T T T T T T T T T T T T T T T T T T T T T T T T T T T T T T T T T T T T T T T T T    
695	. . . . . . . . . . . . G . G G G . G G G G G G G G G G G G G G G G G G G G G G G G G G G G G G G G G G G G G G G G G G G G G G G G G G G G G G G G G    
696	. . . . . . . . . . . . A . A A A . A A A A A A A A A A A A A A A A A A A A A A A A A A A A A A A A A A A A A A A A A A A A A A A A A A A A A A A A A    
697	. . . . . . . . . . . . T . T T T . T T T T T T T T T T T T T T T T T T T T T T T T T T T T T T T T T T T T T T T T T T T T T T T T T T T T T T T T T    
698	. . . . . . . . . . . . C . C . C . C C C C C C C C C C C C C C C C C C C C C C C C C C C C C C C C C C C C C C C C C C C C C C C C C C C C C C C C C    
699	. . . . . . . . . . . . G . G . G . G G G G G G G G G G G G G G G G G G G G G G G G G G G G G G G G G G G G G G G G G G G G G G G G G G G G G G G G G    
700	. . . . . . . . . . . . C . C . C . C C C C C C C C C C C C C C C C C C C C C C C C C C C C C C C C C C C C C C C C C C C C C C C C C C C C C C C C C    
701	. . . . . . . . . . . . A . A . A . A A A A A A A A A A A A A A A A A A A A A A A A A A A A A A A A A A A A A A A A A A A A A A A A A A A A A A A A A    
702	. . . . . . . . . . . . G . G . G . G G G G G G G G G G G G G G G G G G G G G G G G G G G G G G G G G G G G G G G G G G G G G G G G G G G G G G G G G    
703	. . . . . . . . . . . . G . G . G . G G G G G G G G G G G G G G G G G G G G G G G G G G G G G G G G G G G G G G G G G G G G G G G G G G G G G G G G G    
704	. . . . . . . . . . . . C . C . C . C C C C C C C C C C C C C C C C C C C C C C C C C C C C C C C C C C C C C C C C C C C C C C C C C C C C C C C C C    
705	. . . . . . . . . . . . A . A . A . C A A A A A A A A A A A A A A A A A A A A A A A A A A A A A A A A A A A A A A A A A A A A A A A A A A A A A A A A    
706	. . . . . . . . . . . . T . T . T . . T T T T T T T T T T T T T T T T T T T T T T T T T T T T T T T T T T T T T T T T T T T T T T T T T T T T T T T T    
707	. . . . . . . . . . . . T . T . T . . T T T T T T T T T T T T T T T T T T T T T T T T T T T T T T T T T T T T T T T T T T T T T T T T T T T T T T T T    
708	. . . . . . . . . . . . C . C . C . . C C C C C C C C C C C C C C C C C C C C T C C C C C C C C C C C C C C C C C C C C C C C C C C C C C C C C C C C    
709	. . . . . . . . . . . . T . T . . . . T T T T T T T T T T T T T T T T T T T T T T T T T T T T T T T T T T T T T T T T T T T T T T T T T T T T T T T T    
710	. . . . . . . . . . . . T . T . . . . T T T T T T T T T T T T T T T T T T T T T T T T T T T T T T T T T T T T T T T T T T T T T T T T T T T T T T T T    
711	. . . . . . . . . . . . G . G . . . . G G G G G G G G G G G G G G G G G G G G G G G G G G G G G G G G G G G G G G G G G G G G G G G G G G G G G G G G    
712	. . . . . . . . . . . . G . C . . . . G G G G G G G G G G G G G G G G G G G G G G G G G G G G G G G G G G G G G G G G G G G G G G G G G G G G G G G G    
713	. . . . . . . . . . . . C . . . . . . C C C C C C C C C C C C C C C C C C C C C C C C C C C C C C C C C C C C C C C C C C C C C C C C C C C C C C C C    
714	. . . . . . . . . . . . . . . . . . . A A A A A A A A A A A A A A A A A A A A A A A A A A A A A A A A A A A A A A A A A A A A A A A A A A A A A A A A    
715	. . . . . . . . . . . . . . . . . . . T T T T T T T T T T T T T T T T T T T T T T T T T T T T T T T T T T T T T T T T T T T T T T T T T T T T T T T T    
716	. . . . . . . . . . . . . . . . . . . C C C C C C C C C C C C C C C C C C C C C C C C C C C C C C C C C C C C C C C C C C C C C C C C C C C C C C C C    
717	. . . . . . . . . . . . . . . . . . . A A A A A A A A A A A A A A A A A A A A A A A A A A A A A A A A A A A A A A A A A A A A A A A A A A A A A A A A    
718	. . . . . . . . . . . . . . . . . . . A A A A A A A A A A A A A A A A A A A A A A A A A A A A A A A A A A A A A A A A A A A A A A A A A A A A A A A A    
719	. . . . . . . . . . . . . . . . . . . A A A A A A A A A A A A A A A A A A A A A A A A A A A A A A A A A A A A A A A A A A A A A A A A A A A A A A A A    
720	. . . . . . . . . . . . . . . . . . . G G G G G G G G G G G G G G G G G G G G G G G G G G G G G G G G G G G G G G G G G G G G G G G G G G G G G G G G    
721	. . . . . . . . . . . . . . . . . . . A A A A A A A A A A A A A A A A A A A A A A A A A A A A A A A A A A A A A A A A A A A A A A A A A A A A A A A A    
722	. . . . . . . . . . . . . . . . . . . T T T T T T T T T T T T T T T T T T T T T T T T T T T T T T T T T T T T T T T T T T T T T T T T T T T T T T T T    
723	. . . . . . . . . . . . . . . . . . . G G G G G G G G G G G G G G G G G G G G G G G G G G G G G G G G G G G G G G G G G G G G G G G G G G G G G G G G    
724	. . . . . . . . . . . . . . . . . . . T T T T T T T T T T T T T T T T T T T T T T T T T T T T T T T T T T T T T T T T T T T T T T T T T T T T T T T T    
725	. . . . . . . . . . . . . . . . . . . C C C C C C C C C C C C C C C C C C C C C C C C C C C C C C C C C C C C C C C C C C C C C C C C C C C C C C C C    
726	. . . . . . . . . . . . . . . . . . . C C C C C C C C C C C C C C C C C C C C C C C C C C C C C C C C C C C C C C C C C C C C C C C C C C C C C C C C    
727	. . . . . . . . . . . . . . . . . . . T T T T T T T T T T T T T T T T T T T T T T T T T T T T T T T T T T T T T T T T T T T T T T T T T T T T T T T T    
728	. . . . . . . . . . . . . . . . . . . C C C C C C C C C C G C C C C C C C C C T C C C C C C C C C C C C C C C C C C C C C C C C C C C C C C C C C C C    
729	. . . . . . . . . . . . . . . . . . . G G G G G G G G G G . G G G G G G G G G G G G G G G G G G G G G G G G G G G G G G G G G G G G G G G G G G G G G    
730	. . . . . . . . . . . . . . . . . . . T T T T T T T T T T . T T T T T T T T T T T T T T T T T T T T T T T T T T T T T T T T T T T T T T T T T T T T T    
731	. . . . . . . . . . . . . . . . . . . A A A A . A A A A A . A A A A A A A A A A A A A A A A A A A A A A A A A A A A A A A A A A A A A A A A A A A A A    
732	. . . . . . . . . . . . . . . . . . . G G G G . G G G G G . G G G G G G G G G G G G G G G G G G G G G G G G G G G G G G G G G G G G G G G G G G G G G    
733	. . . . . . . . . . . . . . . . . . . A A A A . A A A A A . A A A A A A A A A A A A A A A A A A A A A A A A A A A A A A A A A A A A A A A A A A A A A    
734	. . . . . . . . . . . . . . . . . . . C C C C . C C C C C . C C C C C C C C C C C C C C C C C C C C C C C C C C C C C C C C C C C C C C C C C C C C C    
735	. . . . . . . . . . . . . . . . . . . C C C C . C C C C C . C C C C C C C C C C C C C C C C C C C C C C C C C C C C C C C C C C C C C C C C C C C C C    
736	. . . . . . . . . . . . . . . . . . . A A A A . A A A A A . A A A A A A A A A A A A A A A A A A A A A A A A A A A A A A A A A A A A A A A A A A A A A    
737	. . . . . . . . . . . . . . . . . . . C C C C . C C C C C . . C C C C C C C C C C C C C C C C C C C C C C C C C C C C C C C C C C C C C C C C C C C C    
738	. . . . . . . . . . . . . . . . . . . A A A A . A A A A A . . A A A A A A A A A A A A A A A A A A A A A A A A A A A A A A A A A A A A A A A A A A A A    
739	. . . . . . . . . . . . . . . . . . . T T T T . T T T T T . . T T T T T T T T T T T T T T T T T T T T T T T T T T T T T T T T T T T T T T T T T T T T    
740	. . . . . . . . . . . . . . . . . . . A A A A . A A A . A . . A A A A A A A A A A A A A A A A A A A A A A A A A A A A A A A A A A A A A A A A A A A A    
741	. . . . . . . . . . . . . . . . . . . A A A A . A A A . A . . A A A A A A A A A A A A A A A A A A A A A A A A A A A A A A A A A A A A A A A G A A A A    
742	. . . . . . . . . . . . . . . . . . . G G G G . G G G . G . . G G G G G G G G G G G G G G G G G G G G G G G G G G G G G G G G G G G G G G G G G G G G    
743	. . . . . . . . . . . . . . . . . . . C C C C . C C C . C . . C C C C C C C C C C C C C C C C C C C C C C C C C C C C C C C C C C C C C C C C C C C C    
744	. . . . . . . . . . . . . . . . . . . T T T T . T T T . T . . T T T T T T T T T T T T T T T T T T T T T T T T T T T T T T T T T T T T T T T T T T T T    
745	. . . . . . . . . . . . . . . . . . . G G G G . G G G . G . . G G G G G G G G G G G G G G G G G G G G G G G G G G G G G G G G G G G G G G G G G G G G    
746	. . . . . . . . . . . . . . . . . . . T T T T . T T T . T . . T T T T T T T T T T T T T T T T T T T T T T T T T T T T T T T T T T T T T T T T T T T T    
747	. . . . . . . . . . . . . . . . . . . C C C C . C C C . C . . C C C C C C C C C C C C C C C C C C C C C C C C C C C C C C C C C C C C C C C C C C C C    
748	. . . . . . . . . . . . . . . . . . . C C C C . C C C . C . . C C C C C C C C C C C C C C C C C C C C C C C C C C C C C C C C C C C C C C C C C C C C    
749	. . . . . . . . . . . . . . . . . . . C C C C . C C C . C . . C C C C C C C C C C C C C C C C C C C C C C C C C C C C C C C C C C C C C C C C C C C C    
750	. . . . . . . . . . . . . . . . . . . T T T T . T T T . T . . T T T T T T T T T T T T T T T T T T T T T T T T T T T T T T T T T T T T T T T T T T T T    
751	. . . . . . . . . . . . . . . . . . . C C C C . C C C . C . . C C C C C C C C C C C C C C C C C C C C C C C C C C C C C C C C C C C C C C C C C C C C    
752	. . . . . . . . . . . . . . . . . . . T T T T . T T T . T . . T T T T T T T T T T T T T T T T T T T T T T T T T T T T T T T T T T T T T T T T T T T T    
753	. . . . . . . . . . . . . . . . . . . T T T T . T T T . T . . T T T T T T T T T T T T T T T T T T T T T T T T T T T T T T T T T T T T T T T T T T T T    
754	. . . . . . . . . . . . . . . . . . . G G G G . G G G . G . . G G G G G G G G G G G G G G G G G G G G G G G G G G G G G G G G G G G G G G G G G G G G    
755	. . . . . . . . . . . . . . . . . . . A A A A . A A . . A . . A A A A A A A A A A A A A A A A A A A A A A A A A A A A A A A A A A A A A A A A A A A A    
756	. . . . . . . . . . . . . . . . . . . T T T T . . T . . T . . T T T T T T T T T T T T T T T T T T T T T T T T T T T T T T T T T T T T T T T T T T T T    
757	. . . . . . . . . . . . . . . . . . . T T T T . . T . . T . . T T T T T T T T T T T T T T T T T T T T T T T T T T T T T T T T T T T T T T T T T T T T    
758	. . . . . . . . . . . . . . . . . . . C C C C . . C . . C . . C C C C C C C C C C C C C C C C C C C C C C C C C C C C C C C C C C C C C C C C C C C C    
759	. . . . . . . . . . . . . . . . . . . C C C C . . C . . C . . . C C C C C C C C C C C C C C C C C C C C C C C C C C C C C C C C C C C C C C C C C C C    
760	. . . . . . . . . . . . . . . . . . . G G G G . . G . . G . . . G G G G G G G G G G G G G G G G G G G G G G G G G G G G G G G G G G G G G G G G G G G    
761	. . . . . . . . . . . . . . . . . . . G C C . . . G . . G . . . G G G G G G G G G G G G G G G G G G G G G G G G G G G G G G G G G G G G G G G G G G G    
762	. . . . . . . . . . . . . . . . . . . G . . . . . C . . T . . . C T T T T T T T T T T T T T T T T T T T T T T T T T T T T T T T T T T T T T T T T T T    
763	. . . . . . . . . . . . . . . . . . . . . . . . . G . . G . . . G G G G G G G G G G G G G G G G G G G G G G G G G G G G G G G G G G G G G G G G G G G    
764	. . . . . . . . . . . . . . . . . . . . . . . . . T . . T . . . T T T T T T T T T T T T T T T T T T T T T T T T T T T T T T T T T T T T T T T T T T T    
765	. . . . . . . . . . . . . . . . . . . . . . . . . G . . G . . . G G G G G G G G G G G G G G G G G G G G G G G G G G G G G G G G G G G G G G G G G G G    
766	. . . . . . . . . . . . . . . . . . . . . . . . . T . . T . . . T T T T T T T T T T T T T T T T T T T T T T T T T T T T T T T T T T T T T T T T T T T    
767	. . . . . . . . . . . . . . . . . . . . . . . . . T . . T . . . T T T T T T T T T T T T T T T T T T T T T T T T T T T T T T T T T T T T T T T T T T T    
768	. . . . . . . . . . . . . . . . . . . . . . . . . T . . T . . . T T T T T T T T T T T T T T T T T T T T T T T T T T T T T T T T T T T T T T T T T T T    
769	. . . . . . . . . . . . . . . . . . . . . . . . . C . . C . . . C C C C C C C C C C C C C C C C C C C C C C C C C C C C C C C C C C C C C C C C C C C    
770	. . . . . . . . . . . . . . . . . . . . . . . . . C . . C . . . C C C C C C C C C C C C C C C C C C C C C C C C C C C C C C C C C C C C C C C C C C C    
771	. . . . . . . . . . . . . . . . . . . . . . . . . A . . A . . . A A A A A A A A A A A A A A A A A A A A A A A A A A A A A A A A A A A A A A A A A A A    
772	. . . . . . . . . . . . . . . . . . . . . . . . . G . . G . . . G G G G G G G G G G G G G G G G G G G G G G G G G G G G G G G G G G G G G G G G G G G    
773	. . . . . . . . . . . . . . . . . . . . . . . . . T . . . . . . T T T T T T T T T T T T T T T T T T T T T T T T T T T T T T T T T T T T T T T T T T T    
774	. . . . . . . . . . . . . . . . . . . . . . . . . T . . . . . . T T T T T T T T T T T T T T T T T T T T T T T T T T T T T T T T T T T T T T T T T T T    
775	. . . . . . . . . . . . . . . . . . . . . . . . . C . . . . . . C C C C C C C C C C C C C C C C C C C C C C C C C C C C C C C C C C C C C C C C C C C    
776	. . . . . . . . . . . . . . . . . . . . . . . . . G . . . . . . G G G G G G G G G G G G G G G G G G G G G G G G G G G G G G G G G G G G G G G G G G G    
777	. . . . . . . . . . . . . . . . . . . . . . . . . A . . . . . . A A A A A A A A A A A A A A A A A A A A A A A A A A A A A A A A A A A A A A A A A A A    
778	. . . . . . . . . . . . . . . . . . . . . . . . . A . . . . . . A A A A A A A A A A A A A A A A A A A A A A A A A A A A A A A A A A A A A A A A A A A    
779	. . . . . . . . . . . . . . . . . . . . . . . . . A . . . . . . T T T T T T T T T T T T T T T T T T T T T T T T T T T T T T T T T T T T T T T T T T T    
780	. . . . . . . . . . . . . . . . . . . . . . . . . . . . . . . . C C C C C C C C C C C C C C C C C C C C C C C C C C C C C C C C C C C C C C C C C C C    
781	. . . . . . . . . . . . . . . . . . . . . . . . . . . . . . . . T T T T T T T T T T T T T T T T T T T T T T T T T T T T T T T T T T T T T T T T T T T    
782	. . . . . . . . . . . . . . . . . . . . . . . . . . . . . . . . G G G G G G G G G G G G G G G G G G G G G G G G G G G G G G G G G G G G G G G G G G G    
783	. . . . . . . . . . . . . . . . . . . . . . . . . . . . . . . . C C C C C C C C C C C C C C C C C C C C C C C C C C C C C C C C C C C C C C C C C C C    
784	. . . . . . . . . . . . . . . . . . . . . . . . . . . . . . . . C C C C C C C C C C C C C C C C C C C C C C C C C C C C C C C C C C C C C C C C C C C    
785	. . . . . . . . . . . . . . . . . . . . . . . . . . . . . . . . G G G G G G G G G G G G G G G G G G G G G G G G G G G G G G G G G G G G G G G G G G G    
786	. . . . . . . . . . . . . . . . . . . . . . . . . . . . . . . . T T T T T T T T T T T T T T T T T T T T T T T T T T T T T T T T T T T T T T T T T T T    
787	. . . . . . . . . . . . . . . . . . . . . . . . . . . . . . . . A A A A A A A A A A A A A A A A A A A A A A A A A A A A A A A A A A A A A A A A A A A    
788	. . . . . . . . . . . . . . . . . . . . . . . . . . . . . . . . T T T T T T T T T T T T T T T T T T T T T T T T T T T T T T T T T T T T T T T T T T T    
789	. . . . . . . . . . . . . . . . . . . . . . . . . . . . . . . . T T T T T T T T T T T T T T T T T T T T T T T T T T T T T T T T T T T T T T T T T T T    
790	. . . . . . . . . . . . . . . . . . . . . . . . . . . . . . . . G G G G G G G G G G G G G G G G G G G G G G G G G G G G G G G G G G G G G G G G G G G    
791	. . . . . . . . . . . . . . . . . . . . . . . . . . . . . . . . G G G G G G G G G G G G G G G G G G G G G G G G G G G G G G G G G G G G G G G G G G G    
792	. . . . . . . . . . . . . . . . . . . . . . . . . . . . . . . . T . T T T T T T T T T T T T T T T T T T T T T T T T T T T T T T T T T T T T T T T T T    
793	. . . . . . . . . . . . . . . . . . . . . . . . . . . . . . . . C . C C C C C C C C C C C C C C C C C C C C C C C C C C C C C C C C C C C C C C C C C    
794	. . . . . . . . . . . . . . . . . . . . . . . . . . . . . . . . C . C C C C C C C C C C C C C C C C C C C C C C C C C C C C C C C C C C C C C C C C C    
795	. . . . . . . . . . . . . . . . . . . . . . . . . . . . . . . . A . A A A A A A A A A A A A A A A A A A A A A A A A A A A A A A A A A A A A A A A A A    
796	. . . . . . . . . . . . . . . . . . . . . . . . . . . . . . . . T . T T T T T T T T T T T T T T T T T T T T T T T T T T T T T T T T T T T T T T T T T    
797	. . . . . . . . . . . . . . . . . . . . . . . . . . . . . . . . A . A A A A A A A A A A A A A A A A A A A A A A A A A A A A A A A A A A A A A A A A A    
798	. . . . . . . . . . . . . . . . . . . . . . . . . . . . . . . . C . C C C C C C C C C C C C C C C C C C C C C C C C C C C C C C C C C C C C C C C C C    
799	. . . . . . . . . . . . . . . . . . . . . . . . . . . . . . . . T . T T T T T T T T T T T T T T T T T T T T T T T T T T T T T T T T T T T T T T T T T    
800	. . . . . . . . . . . . . . . . . . . . . . . . . . . . . . . . T . T T T T T T T T T T T T T T T T T T T T T T T T T T T T T T T T T T T T T T T T T    
801	. . . . . . . . . . . . . . . . . . . . . . . . . . . . . . . . C . C C C C C C C C C C C C C C C C C C C C C C C C C C C C C C C C C C C C C C C C C    
802	. . . . . . . . . . . . . . . . . . . . . . . . . . . . . . . . C . C C C C C C C C C C C C C C C C C C C C C C C C C C C C C C C C C C C C C C C C C    
803	. . . . . . . . . . . . . . . . . . . . . . . . . . . . . . . . C . C C C C C C C C C C C C C C C C C C C C C C C C C C C C C C C C C C C C C C C C C    
804	. . . . . . . . . . . . . . . . . . . . . . . . . . . . . . . . A . A A A A A A A A A A A A A A A A A A A A A A A A A A A A A A A A A A A A A A A A A    
805	. . . . . . . . . . . . . . . . . . . . . . . . . . . . . . . . A . A A A A A A A A A A A A A A A A A A A A A A A A A A A A A A A A A A A A A A A A A    
806	. . . . . . . . . . . . . . . . . . . . . . . . . . . . . . . . A . A A A A A A A A A A A A A A A A A A A A A A A A A A A A A A A A A A A A A A A A A    
807	. . . . . . . . . . . . . . . . . . . . . . . . . . . . . . . . G . G G G G G G G G G G G G G G G G G G G G G G G G G G G G G G G G G G G G G G G G G    
808	. . . . . . . . . . . . . . . . . . . . . . . . . . . . . . . . A . A A A A A A A A A A A A A A A A A A A A A A A A A A A A A A A A A A A A A A A A A    
809	. . . . . . . . . . . . . . . . . . . . . . . . . . . . . . . . T . T T T T T T T T T T T T T T T T T T T T T T T T T T T T T T T T T T T T T T T T T    
810	. . . . . . . . . . . . . . . . . . . . . . . . . . . . . . . . G . G . G G G G G G G G G G G G G G G G G G G G G G G G G G G G G G G G G G G G G G G    
811	. . . . . . . . . . . . . . . . . . . . . . . . . . . . . . . . T . T . T T T T T T T T T T T T T T T T T T T T T T T T T T T T T T T T T T T T T T T    
812	. . . . . . . . . . . . . . . . . . . . . . . . . . . . . . . . C . C . C C C C C C C C C C C C C C C C C C C C C C C C C C C C C C C C C C C C C C C    
813	. . . . . . . . . . . . . . . . . . . . . . . . . . . . . . . . A . A . A A A A A A A A A A A A A A A A A A A A A A A A A A A A A A A A A A A A A A A    
814	. . . . . . . . . . . . . . . . . . . . . . . . . . . . . . . . T . T . T T T T T T T T T T T T T T T T T T T T T T T T T T T T T T T T T T T T T T T    
815	. . . . . . . . . . . . . . . . . . . . . . . . . . . . . . . . A . A . A A A A A A A A A A A A A A A A A A A A A A A A A A A A A A A A A A A A A A A    
816	. . . . . . . . . . . . . . . . . . . . . . . . . . . . . . . . C . C . C C C C C C C C C C C C C C C C C C C C C C C C C C C C C C C C C C C C C C C    
817	. . . . . . . . . . . . . . . . . . . . . . . . . . . . . . . . C . A . A A A A A A A A A A A A A A A A A A A A A A A A A A A A A A A A A A A A A A A    
818	. . . . . . . . . . . . . . . . . . . . . . . . . . . . . . . . . . T . T T T T T T T T T T T T T T T T T T T T T T T T T T T T T T T T T T T T T T T    
819	. . . . . . . . . . . . . . . . . . . . . . . . . . . . . . . . . . T . T T T T T T T T T T T T T T T T T T T T T T T T T T T T T T T T T T T T T T T    
820	. . . . . . . . . . . . . . . . . . . . . . . . . . . . . . . . . . T . T T T T T T T T T T T T T T T T T T T T T T T T T T T T T T T T T T T T T T T    
821	. . . . . . . . . . . . . . . . . . . . . . . . . . . . . . . . . . T . C C C C C C C C C C C C C C C C C C C C C C C C C C C C C C C C C C C C C C C    
822	. . . . . . . . . . . . . . . . . . . . . . . . . . . . . . . . . . . . - . - - A - - - - - - - - - - - - - - - - - - - - - - - - - - - - - - - - - -    
823	. . . . . . . . . . . . . . . . . . . . . . . . . . . . . . . . . . . . C . C C C C C C C C C C C C C C C C C C C C C C C C C C C C C C C C C C C C C    
824	. . . . . . . . . . . . . . . . . . . . . . . . . . . . . . . . . . . . T . T T T T T T T T T T T T T T T T T T T T T T T T T T T T T T T T T T T T T    
825	. . . . . . . . . . . . . . . . . . . . . . . . . . . . . . . . . . . . C . C C C C C C C C C C C C C C C C C C C C C C C C C C C C C C C C C C C C C    
826	. . . . . . . . . . . . . . . . . . . . . . . . . . . . . . . . . . . . A . A A A A A A A A A A A A A A A A A A A A A A A A A A A A A A A A A A A A A    
827	. . . . . . . . . . . . . . . . . . . . . . . . . . . . . . . . . . . . G . G G G G G G G G G G G G G G G G G G G G G G G G G G G G G G G G G G G G G    
828	. . . . . . . . . . . . . . . . . . . . . . . . . . . . . . . . . . . . C . C C C C C C C C C C C C C C C C C C C C C C C C C C C C C C C C C C C C C    
829	. . . . . . . . . . . . . . . . . . . . . . . . . . . . . . . . . . . . T . T T T T T T T T T T T T T T T T T T T T T T T T T T T T T T T T G T T T T    
830	. . . . . . . . . . . . . . . . . . . . . . . . . . . . . . . . . . . . G . G G G G G G G G G G G G G G G G G G G G G G G G G G G G G G G G G G G G G    
831	. . . . . . . . . . . . . . . . . . . . . . . . . . . . . . . . . . . . T . T T T T T T T T T T T T T T T T T T T T T T T T T T T T T T T T T T T T T    
832	. . . . . . . . . . . . . . . . . . . . . . . . . . . . . . . . . . . . G . G G G G G G G G G G G G G G G G G G G G G G G G G G G G G G G G G G G G G    
833	. . . . . . . . . . . . . . . . . . . . . . . . . . . . . . . . . . . . A . A A A A A A A A A A A A A A A A A A A A A A A A A A A A A A A A A A A A A    
834	. . . . . . . . . . . . . . . . . . . . . . . . . . . . . . . . . . . . T . T T T T T T T T T T T T T T T T T T T T T T T T T T T T T T T T T T T T T    
835	. . . . . . . . . . . . . . . . . . . . . . . . . . . . . . . . . . . . C . C C C C C C C C C C C C C C C C C C C C C C C C C C C C C C C C C C C C C    
836	. . . . . . . . . . . . . . . . . . . . . . . . . . . . . . . . . . . . . . T T T T T T T T T T T T T T T T T T T T T T T T T T T T T T T T T T T T T    
837	. . . . . . . . . . . . . . . . . . . . . . . . . . . . . . . . . . . . . . T T T T T T T T T T T T T T T T T T T T T T T T T T T T T T T T T T T T T    
838	. . . . . . . . . . . . . . . . . . . . . . . . . . . . . . . . . . . . . . G G G G G G G G G G G G G G G G G G G G G G G G G G G G G G G G G G G G G    
839	. . . . . . . . . . . . . . . . . . . . . . . . . . . . . . . . . . . . . . T T T T T T T T T T T T T T T T T T T T T T T T T T T T T T T T T T T T T    
840	. . . . . . . . . . . . . . . . . . . . . . . . . . . . . . . . . . . . . . A A A A A A A A A A A A A A A A A A A A A A A A A A A A A A A A A A A A A    
841	. . . . . . . . . . . . . . . . . . . . . . . . . . . . . . . . . . . . . . T T T T T T T T T T T T T T T T T T T T T T T T T T T T T T T T T T T T T    
842	. . . . . . . . . . . . . . . . . . . . . . . . . . . . . . . . . . . . . . . G G G G G G G G G G G G G G G G G G G G G G G G G G G G G G G G G G G G    
843	. . . . . . . . . . . . . . . . . . . . . . . . . . . . . . . . . . . . . . . G G G G G G G G G G G G G G G G G G G G G G G G G G G G G G G G G G G G    
844	. . . . . . . . . . . . . . . . . . . . . . . . . . . . . . . . . . . . . . . T T T T T T T T T T T T T T T T T T T T T T T T T T T T T T T T T T T T    
845	. . . . . . . . . . . . . . . . . . . . . . . . . . . . . . . . . . . . . . . A A A A A A A A A A A A A A A A A A A A A A A A A A A A A A A A A A A A    
846	. . . . . . . . . . . . . . . . . . . . . . . . . . . . . . . . . . . . . . . G G G G G G G G G G G G G G G G G G G G G G G G G G G G G G G G G G G G    
847	. . . . . . . . . . . . . . . . . . . . . . . . . . . . . . . . . . . . . . . A A A A A A A A A A A A A A A A A A A A A A A A A A A A A A A A A A A A    
848	. . . . . . . . . . . . . . . . . . . . . . . . . . . . . . . . . . . . . . . T T T T T T T T T T T T T T T T T T T T T T T T T T T T T T T T T T T T    
849	. . . . . . . . . . . . . . . . . . . . . . . . . . . . . . . . . . . . . . . T T T T T T T T T T T T T T T T T T T T T T T T T T T T T T T T T T T T    
850	. . . . . . . . . . . . . . . . . . . . . . . . . . . . . . . . . . . . . . . T T T T T T T T T T T T T T T T T T T T T T T T T T T T T T T T T T T T    
851	. . . . . . . . . . . . . . . . . . . . . . . . . . . . . . . . . . . . . . . C C C C C C C C C C C C C C C C C C C C C C C C C C C C C C C C C C C C    
852	. . . . . . . . . . . . . . . . . . . . . . . . . . . . . . . . . . . . . . . T T T T T T T T T T T T T T T T T T T T T T T T T T T T T T T T T T T T    
853	. . . . . . . . . . . . . . . . . . . . . . . . . . . . . . . . . . . . . . . A A A A A A A A A A A A A A A A A A A A A A A A A A A A A A A A A A A A    
854	. . . . . . . . . . . . . . . . . . . . . . . . . . . . . . . . . . . . . . . A A A A A A A A A A A A A A A A A A A A A A A A A A A A A A A A A A A A    
855	. . . . . . . . . . . . . . . . . . . . . . . . . . . . . . . . . . . . . . . T T T T T T T T T T T T T T T T T T T T T T T T T T T T T T T T T T T T    
856	. . . . . . . . . . . . . . . . . . . . . . . . . . . . . . . . . . . . . . . G G G G G G G G G G G G G G G G G G G G G G G G G G G G G G G G G G G G    
857	. . . . . . . . . . . . . . . . . . . . . . . . . . . . . . . . . . . . . . . T T T T T T T T T T T T T T T T T T T T T T T T T T T T T T T T T T T T    
858	. . . . . . . . . . . . . . . . . . . . . . . . . . . . . . . . . . . . . . . A A A A A A A A A A A A A A A A A A A A A A A A A A A A A A A A A A A A    
859	. . . . . . . . . . . . . . . . . . . . . . . . . . . . . . . . . . . . . . . G G G G G G G G G G G G G G G G G G G G G G G G G G G G G G G G G G G G    
860	. . . . . . . . . . . . . . . . . . . . . . . . . . . . . . . . . . . . . . . A A A A A A A A A A A A A A A A A A A A A A A A A A A A A A A A A A A A    
861	. . . . . . . . . . . . . . . . . . . . . . . . . . . . . . . . . . . . . . . C G G G G G G G G G G G G G G G G G G G G G G G G G G G G G G G G G G G    
862	. . . . . . . . . . . . . . . . . . . . . . . . . . . . . . . . . . . . . . . . T T T T T T T T T T T T T T T T T T T T T T T T T T T T T T T T T T T    
863	. . . . . . . . . . . . . . . . . . . . . . . . . . . . . . . . . . . . . . . . A A A A A A A A A A A A A A A A A A A A A A A A A A A A A A A A A A A    
864	. . . . . . . . . . . . . . . . . . . . . . . . . . . . . . . . . . . . . . . . T T T T T T T T T T T T T T T T T T T T T T T T T T T T T T T T T T T    
865	. . . . . . . . . . . . . . . . . . . . . . . . . . . . . . . . . . . . . . . . T T T T T T T T T T T T T T T T T T T T T T T T T T T T T T T T T T T    
866	. . . . . . . . . . . . . . . . . . . . . . . . . . . . . . . . . . . . . . . . C C C C C C C C C C C C C C C C C C C C C C C C C C C C C C C C C C C    
867	. . . . . . . . . . . . . . . . . . . . . . . . . . . . . . . . . . . . . . . . T T T T T T T T T T T T T T T T T T T T T T T T T T T T T T T T T T T    
868	. . . . . . . . . . . . . . . . . . . . . . . . . . . . . . . . . . . . . . . . G G G G G G G G G G G G G G G G G G G G G G G G G G G G G G G G G G G    
869	. . . . . . . . . . . . . . . . . . . . . . . . . . . . . . . . . . . . . . . . T T T T T T T T T T T T T T T T T T T T T T T T T T T T T T T T T T T    
870	. . . . . . . . . . . . . . . . . . . . . . . . . . . . . . . . . . . . . . . . T T T T T T T T T T T T T T T T T T T T T T T T T T T T T T T T T T T    
871	. . . . . . . . . . . . . . . . . . . . . . . . . . . . . . . . . . . . . . . . G G G G G G G G G G G G G G G G G G G G G G G G G G G G G G G G G G G    
872	. . . . . . . . . . . . . . . . . . . . . . . . . . . . . . . . . . . . . . . . A A A A A A A A A A A A A A A A A A A A A A A A A A A A A A A A A A A    
873	. . . . . . . . . . . . . . . . . . . . . . . . . . . . . . . . . . . . . . . . C C C C C C C C C C C C C C C C C C C C C C C C C C C C C C C C C C C    
874	. . . . . . . . . . . . . . . . . . . . . . . . . . . . . . . . . . . . . . . . T T T T T T T T T T T T T T T T T T T T T T T T T T T T T T T T T T T    
875	. . . . . . . . . . . . . . . . . . . . . . . . . . . . . . . . . . . . . . . . T T T T T T T T T T T T T T T T T T T T T T T T T T T T T T T T T T T    
876	. . . . . . . . . . . . . . . . . . . . . . . . . . . . . . . . . . . . . . . . C C C C C C C C C C C C C C C C C C C C C C C C C C C C C C C C C C C    
877	. . . . . . . . . . . . . . . . . . . . . . . . . . . . . . . . . . . . . . . . A A A A A A A A A A A A A A A A A A A A A A A A A A A A A A A A A A A    
878	. . . . . . . . . . . . . . . . . . . . . . . . . . . . . . . . . . . . . . . . G G G G G G G G G G G G G G G G G G G G G G G G G G G G G G G G G G G    
879	. . . . . . . . . . . . . . . . . . . . . . . . . . . . . . . . . . . . . . . . G G G G G G G G G G G G G G G G G G G G G G G G G G G G G G G G G G G    
880	. . . . . . . . . . . . . . . . . . . . . . . . . . . . . . . . . . . . . . . . T T T T T T T T T T T T C T T T T T T T T T T T T T T T T T T T T T T    
881	. . . . . . . . . . . . . . . . . . . . . . . . . . . . . . . . . . . . . . . . G G G G G G G G G G G G G G G G G G G G G G G G G G G G G G G G G G G    
882	. . . . . . . . . . . . . . . . . . . . . . . . . . . . . . . . . . . . . . . . G G G G G G G G G G G G G G G G G G G G G G G G G G G G G G G G G G G    
883	. . . . . . . . . . . . . . . . . . . . . . . . . . . . . . . . . . . . . . . . T T T T T T T T T T T T T T T T T T T T T T T T T T T T T T T T T T T    
884	. . . . . . . . . . . . . . . . . . . . . . . . . . . . . . . . . . . . . . . . A A A A A A A A G A A A A A A A A A A A A A A A A A A A A A A A A A A    
885	. . . . . . . . . . . . . . . . . . . . . . . . . . . . . . . . . . . . . . . . A A A A A A A A A A A A A A A A A A A A A A A A A A A A A A A A A A A    
886	. . . . . . . . . . . . . . . . . . . . . . . . . . . . . . . . . . . . . . . . T T T T T T T T T T T T T T T T T T T T T T T T T T T T T T T T T T T    
887	. . . . . . . . . . . . . . . . . . . . . . . . . . . . . . . . . . . . . . . . C C C C C C C C C C C C C C C C C C C C C C C C C C C C C C C C C C C    
888	. . . . . . . . . . . . . . . . . . . . . . . . . . . . . . . . . . . . . . . . G G G G G G G G G G G G G G G G G G G G G G G G G G G G G G G G G G G    
889	. . . . . . . . . . . . . . . . . . . . . . . . . . . . . . . . . . . . . . . . A A A A A A A A A A A A A A A A A A A A A A A A A A A A A A A A A A A    
890	. . . . . . . . . . . . . . . . . . . . . . . . . . . . . . . . . . . . . . . . A A A A A A A A A A A A A A A A A A A A A A A A A A A A A A A A A A A    
891	. . . . . . . . . . . . . . . . . . . . . . . . . . . . . . . . . . . . . . . . T T T T T T T T T T T T T T T T T T T T T T T T T T T T T T T T T T T    
892	. . . . . . . . . . . . . . . . . . . . . . . . . . . . . . . . . . . . . . . . A A A A A A A A A A A A A A A A A A A A A A A A A A A A A A A A A A A    
893	. . . . . . . . . . . . . . . . . . . . . . . . . . . . . . . . . . . . . . . . T T T T T T T T T T T T T T T T T T T T T T T T T T T T T T T T T T T    
894	. . . . . . . . . . . . . . . . . . . . . . . . . . . . . . . . . . . . . . . . T T T T T T T T T T T T T T T T T T T T T T T T T T T T T T T T T T T    
895	. . . . . . . . . . . . . . . . . . . . . . . . . . . . . . . . . . . . . . . . G G G G G G G G G G G G G G G G G G G G G G G G G G G G G G G G G G G    
896	. . . . . . . . . . . . . . . . . . . . . . . . . . . . . . . . . . . . . . . . G G G G G G G G G G G G G G G G G G G G G G G G G G G G G G G G G G G    
897	. . . . . . . . . . . . . . . . . . . . . . . . . . . . . . . . . . . . . . . . C C C C C C C C C C C C C C C C C C C C C C C C C C C C C C C C C C C    
898	. . . . . . . . . . . . . . . . . . . . . . . . . . . . . . . . . . . . . . . . T T T T T T T T T T T T T T T T T T T T T T T T T T T T T T T T T T T    
899	. . . . . . . . . . . . . . . . . . . . . . . . . . . . . . . . . . . . . . . . C C C C C C C C C C C C C C C C C C C C C C C C C C C C C C C C C C C    
900	. . . . . . . . . . . . . . . . . . . . . . . . . . . . . . . . . . . . . . . . G G G G G G G G G G G G G G G G G G G G G G G G G G G G G G G G G G G    
901	. . . . . . . . . . . . . . . . . . . . . . . . . . . . . . . . . . . . . . . . T T T T T T T T T T T T T T T T T T T T T T T T T T T T T T T T T T T    
902	. . . . . . . . . . . . . . . . . . . . . . . . . . . . . . . . . . . . . . . . T T T T T T T T T T T T T T T T T T T T T T T T T T T T T T T T T T T    
903	. . . . . . . . . . . . . . . . . . . . . . . . . . . . . . . . . . . . . . . . T T T T T T T T T T T T T T T T T T T T T T T T T T T T T T T T T T T    
904	. . . . . . . . . . . . . . . . . . . . . . . . . . . . . . . . . . . . . . . . A A A A A A A A A A A A A A A A A A A A A A A A A A A A A A A A A A A    
905	. . . . . . . . . . . . . . . . . . . . . . . . . . . . . . . . . . . . . . . . G G G G G G G G G G G G G G G G G G G G G G G G G G G G G G G G G G G    
906	. . . . . . . . . . . . . . . . . . . . . . . . . . . . . . . . . . . . . . . . C C C C C C C C C C C C C C C C C C C C C C C C C C C C C C C C C C C    
907	. . . . . . . . . . . . . . . . . . . . . . . . . . . . . . . . . . . . . . . . T T T T T T T T T T T T T T T T T T T T T T T T T T T T T T T T T T T    
908	. . . . . . . . . . . . . . . . . . . . . . . . . . . . . . . . . . . . . . . . G G G G G G G G G G G G G G G G G G G G G G G G G G G G G G G G G G G    
909	. . . . . . . . . . . . . . . . . . . . . . . . . . . . . . . . . . . . . . . . C C C C C C C C C C C C C C C C C C C C C C C C C C C C C C C C C C C    
910	. . . . . . . . . . . . . . . . . . . . . . . . . . . . . . . . . . . . . . . . T T T T T T T T T T T T T T T T T T T T T T T T T T T T T T T T T T T    
911	. . . . . . . . . . . . . . . . . . . . . . . . . . . . . . . . . . . . . . . . . T T T T T T T T T T T T T T T T T T T T T T T T T T T T T T T T T T    
912	. . . . . . . . . . . . . . . . . . . . . . . . . . . . . . . . . . . . . . . . . G G G G G G G G G G G G G G G G G G G G G G G G G G G G G G G G G G    
913	. . . . . . . . . . . . . . . . . . . . . . . . . . . . . . . . . . . . . . . . . C C C C C C C C C C C C C C C C C C C C C C C C C C C C C C C C C C    
914	. . . . . . . . . . . . . . . . . . . . . . . . . . . . . . . . . . . . . . . . . A A A A A A A A A A A A A A A A A A A A A A A A A A A A A A A A A A    
915	. . . . . . . . . . . . . . . . . . . . . . . . . . . . . . . . . . . . . . . . . T T T T T T T T T T T T T T T T T T T T T T T T T T T T T T T T T T    
916	. . . . . . . . . . . . . . . . . . . . . . . . . . . . . . . . . . . . . . . . . A A A A A A A A A A A A A A A A A A A A A A A A A A A A A A A A A A    
917	. . . . . . . . . . . . . . . . . . . . . . . . . . . . . . . . . . . . . . . . . G G G G G G G G G G G G G G G G G G G G G G G G G G G G G G G G G G    
918	. . . . . . . . . . . . . . . . . . . . . . . . . . . . . . . . . . . . . . . . . C C C C C C C C C C C C C C C C C C C C C C C C C C C C C C C C C C    
919	. . . . . . . . . . . . . . . . . . . . . . . . . . . . . . . . . . . . . . . . . C C C C C C C C C C C C C C C C C C C C C C C C C C C C C C C C C C    
920	. . . . . . . . . . . . . . . . . . . . . . . . . . . . . . . . . . . . . . . . . A A A A A A A A A A A A A A A A A A A A A A A A A A A A A A A A A A    
921	. . . . . . . . . . . . . . . . . . . . . . . . . . . . . . . . . . . . . . . . . T T T T T T T T T T T T T T T T T T T T T T T T T T T T T T T T T T    
922	. . . . . . . . . . . . . . . . . . . . . . . . . . . . . . . . . . . . . . . . . G G G G G G G G G G G G G G G G G G G G G G G G G G G G G G G G G G    
923	. . . . . . . . . . . . . . . . . . . . . . . . . . . . . . . . . . . . . . . . . C C C C C C C C C C C C C C C C C C C C C C C C C C C C C C C C C C    
924	. . . . . . . . . . . . . . . . . . . . . . . . . . . . . . . . . . . . . . . . . T T T T T T T T T T T T T T T T T T T T T T T T T T T T T T T T T T    
925	. . . . . . . . . . . . . . . . . . . . . . . . . . . . . . . . . . . . . . . . . T T T T T T T T T T T T T T T T T T T T T T T T T T T T T T T T T T    
926	. . . . . . . . . . . . . . . . . . . . . . . . . . . . . . . . . . . . . . . . . G G G G G G G G G G G G G G G G G G G G G G G G G G G G G G G G G G    
927	. . . . . . . . . . . . . . . . . . . . . . . . . . . . . . . . . . . . . . . . . T T T T T T T T T T T T T T T T T T T T T T T T T T T T T T T T T T    
928	. . . . . . . . . . . . . . . . . . . . . . . . . . . . . . . . . . . . . . . . . A A A A A A A A A A A A A A A A A A A A A A A A A A A A A A A A A A    
929	. . . . . . . . . . . . . . . . . . . . . . . . . . . . . . . . . . . . . . . . . T T T T T T T T T T T T T T T T T T T T T T T T T T T T T T T T T T    
930	. . . . . . . . . . . . . . . . . . . . . . . . . . . . . . . . . . . . . . . . . A A A A A A A A A A A A A A A A A A A A A A A A A A A A A A A A A A    
931	. . . . . . . . . . . . . . . . . . . . . . . . . . . . . . . . . . . . . . . . . T T T T T T T T T T T T T T T T T T T T T T T T T T T T T T T T T T    
932	. . . . . . . . . . . . . . . . . . . . . . . . . . . . . . . . . . . . . . . . . T T T T T T T T T T T T T T T T T T T T T T T T T T T T T T T T T T    
933	. . . . . . . . . . . . . . . . . . . . . . . . . . . . . . . . . . . . . . . . . T T T T T T T T T T T T T T T T T T T T T T T T T T T T T T T T T T    
934	. . . . . . . . . . . . . . . . . . . . . . . . . . . . . . . . . . . . . . . . . T T T T T T T T T T T T T T T T T T T T T T T T T T T T T T T T T T    
935	. . . . . . . . . . . . . . . . . . . . . . . . . . . . . . . . . . . . . . . . . G G G G G G G G G G G G G G G G G G G G G G G G G G G G G G G G G G    
936	. . . . . . . . . . . . . . . . . . . . . . . . . . . . . . . . . . . . . . . . . T T T T T T T T T T T T T T T T T T T T T T T T T T T T T T T T T T    
937	. . . . . . . . . . . . . . . . . . . . . . . . . . . . . . . . . . . . . . . . . G G G G G G G G G G G G G G G G G G G G G G G G G G G G G G G G G G    
938	. . . . . . . . . . . . . . . . . . . . . . . . . . . . . . . . . . . . . . . . . T T T T T T T T T T T T T T T T T T T T T T T T T T T T T T T T T T    
939	. . . . . . . . . . . . . . . . . . . . . . . . . . . . . . . . . . . . . . . . . A A A A A A A A A A A A A A A A A A A A A A A A A A A A A A A A A A    
940	. . . . . . . . . . . . . . . . . . . . . . . . . . . . . . . . . . . . . . . . . C C C C C C C C C C C C C C C C C C C C C C C C C C C C C C C C C C    
941	. . . . . . . . . . . . . . . . . . . . . . . . . . . . . . . . . . . . . . . . . A A A A A A A A A A A A A A A A A A A A A A A A A A A A A A A A A A    
942	. . . . . . . . . . . . . . . . . . . . . . . . . . . . . . . . . . . . . . . . . A A A A A A A A A A A A A A A A A A A A A A A A A A A A A A A A A A    
943	. . . . . . . . . . . . . . . . . . . . . . . . . . . . . . . . . . . . . . . . . A A A A A A A A A A A A A A A A A A A A A A A A A A A A A A A A A A    
944	. . . . . . . . . . . . . . . . . . . . . . . . . . . . . . . . . . . . . . . . . A A A A A A A A A A A A A A A A A A A A A A A A A A A A A A A A A A    
945	. . . . . . . . . . . . . . . . . . . . . . . . . . . . . . . . . . . . . . . . . T T T T T T T T T T T T T T T T T T T T T T T T T T T T T T T T T T    
946	. . . . . . . . . . . . . . . . . . . . . . . . . . . . . . . . . . . . . . . . . G G G G G G G G G G G G G G G G G G G G G G G G G G G G G G G G G G    
947	. . . . . . . . . . . . . . . . . . . . . . . . . . . . . . . . . . . . . . . . . T T T T T T T T T T T T T T T T T T T T T T T T T T T T T T T T T T    
948	. . . . . . . . . . . . . . . . . . . . . . . . . . . . . . . . . . . . . . . . . G G G G G G G G G G G G G G G G G G G G G G G G G G G G G G G G G G    
949	. . . . . . . . . . . . . . . . . . . . . . . . . . . . . . . . . . . . . . . . . C C C C C C C C C C C C C C C C C C C C C C C C C C C C C C C C C C    
950	. . . . . . . . . . . . . . . . . . . . . . . . . . . . . . . . . . . . . . . . . T T T T T T T T T T T T T T T T T T T T T T T T T T T T T T T T T T    
951	. . . . . . . . . . . . . . . . . . . . . . . . . . . . . . . . . . . . . . . . . A A A A A A A A A A A A A A A A A A A A A A A A A A A A A A A A A A    
952	. . . . . . . . . . . . . . . . . . . . . . . . . . . . . . . . . . . . . . . . . A A A A A A A A A A A A A A A A A A A A A A A A A A A A A A A A A A    
953	. . . . . . . . . . . . . . . . . . . . . . . . . . . . . . . . . . . . . . . . . G G G G G G G G G G G G G G G G G G G G G G G G G G G G G G G G G G    
954	. . . . . . . . . . . . . . . . . . . . . . . . . . . . . . . . . . . . . . . . . A A A A A A A A A A A A A A A A A A A A A A A A A A A A A A A A A A    
955	. . . . . . . . . . . . . . . . . . . . . . . . . . . . . . . . . . . . . . . . . A A A A A A A A A A A A A A A A A A A A A A A A A A A A A A A A A A    
956	. . . . . . . . . . . . . . . . . . . . . . . . . . . . . . . . . . . . . . . . . A A A A A A A A A A A A A A A A A A A A A A A A A A A A A A A A A A    
957	. . . . . . . . . . . . . . . . . . . . . . . . . . . . . . . . . . . . . . . . . A A A A A A A A A A A A A A A A A A A A A A A A A A A A A A A A A A    
958	. . . . . . . . . . . . . . . . . . . . . . . . . . . . . . . . . . . . . . . . . A A A A A A A A A A A A A A A A A A A A A A A A A A A A A A A A A A    
959	. . . . . . . . . . . . . . . . . . . . . . . . . . . . . . . . . . . . . . . . . . T T T T T T T T T T T T T T T T T T T T T T T T T T T T T T T T T    
960	. . . . . . . . . . . . . . . . . . . . . . . . . . . . . . . . . . . . . . . . . . A A A A A A A A A A A A A A A A A A A A A A A A A A A A A A A A A    
961	. . . . . . . . . . . . . . . . . . . . . . . . . . . . . . . . . . . . . . . . . . T T T T T T T T T T T T T T T T T T T T T T T T T T T T T T T T T    
962	. . . . . . . . . . . . . . . . . . . . . . . . . . . . . . . . . . . . . . . . . . A A A A A A A A A A A A A A A A A A A A A A A A A A A A A A A A A    
963	. . . . . . . . . . . . . . . . . . . . . . . . . . . . . . . . . . . . . . . . . . C C C C C C C C C C C C C C C C C C C C C C C C C C C C C C C C C    
964	. . . . . . . . . . . . . . . . . . . . . . . . . . . . . . . . . . . . . . . . . . G G G G G G G G G G G G G G G G G G G G G G G G G G G G G G G G G    
965	. . . . . . . . . . . . . . . . . . . . . . . . . . . . . . . . . . . . . . . . . . A A A A A A A A A A A A A A A A A A A A A A A A A A A A A A A A A    
966	. . . . . . . . . . . . . . . . . . . . . . . . . . . . . . . . . . . . . . . . . . A A A A A A A A A A A A A A A A A A A A A A A A A A A A A A A A A    
967	. . . . . . . . . . . . . . . . . . . . . . . . . . . . . . . . . . . . . . . . . . A A A A A A A A A A A A A A A A A A A A A A A A A A A A A A A A A    
968	. . . . . . . . . . . . . . . . . . . . . . . . . . . . . . . . . . . . . . . . . . A A A A A A A A A A A A A A A A A A A A A A A A A A A A A A A A A    
969	. . . . . . . . . . . . . . . . . . . . . . . . . . . . . . . . . . . . . . . . . . C C C C C C C C C C C C C C C C C C C C C C C C C C C C C C C C C    
970	. . . . . . . . . . . . . . . . . . . . . . . . . . . . . . . . . . . . . . . . . . T T T T T T T T T T T T T T T T T T T T T T T T T T T T T T T T T    
971	. . . . . . . . . . . . . . . . . . . . . . . . . . . . . . . . . . . . . . . . . . A A A A A A A A A A A A A A A A A A A A A A A A A A A A A A A A A    
972	. . . . . . . . . . . . . . . . . . . . . . . . . . . . . . . . . . . . . . . . . . C C C C C C C C C C C C C C C C C C C C C C C C C C C C C C C C C    
973	. . . . . . . . . . . . . . . . . . . . . . . . . . . . . . . . . . . . . . . . . . T T T T T T T T T T T T T T T T T T T T T T T T T T T T T T T T T    
974	. . . . . . . . . . . . . . . . . . . . . . . . . . . . . . . . . . . . . . . . . . T T T T T T T T T T T T T T T T T T T T T T T T T T T T T T T T T    
975	. . . . . . . . . . . . . . . . . . . . . . . . . . . . . . . . . . . . . . . . . . T T T T T T T T T T T T T T T T T T T T T T T T T T T T T T T T T    
976	. . . . . . . . . . . . . . . . . . . . . . . . . . . . . . . . . . . . . . . . . . T T T T G T T T T T T T T T T T T T T T T T T T T T T T T T T T T    
977	. . . . . . . . . . . . . . . . . . . . . . . . . . . . . . . . . . . . . . . . . . G G G G . G G G G G G G G G G G G G G G G G G G G G G G G G G G G    
978	. . . . . . . . . . . . . . . . . . . . . . . . . . . . . . . . . . . . . . . . . . T T T T . T T T T T T T T T T T T T T T T T T T T T T T T T T T T    
979	. . . . . . . . . . . . . . . . . . . . . . . . . . . . . . . . . . . . . . . . . . C C C C . C C C C C C C C C C C C C C C C C C C C C C C C C C C C    
980	. . . . . . . . . . . . . . . . . . . . . . . . . . . . . . . . . . . . . . . . . . A A A A . A A A A A A A A A A A A A A A A A A A A A A A A A A A A    
981	. . . . . . . . . . . . . . . . . . . . . . . . . . . . . . . . . . . . . . . . . . T T T T . T T T T T T T T T T T T T T T T T T T T T T T T T T T T    
982	. . . . . . . . . . . . . . . . . . . . . . . . . . . . . . . . . . . . . . . . . . C C C C . C C C C C C C C C C C C C C C C C C C C C C C C C C C C    
983	. . . . . . . . . . . . . . . . . . . . . . . . . . . . . . . . . . . . . . . . . . G G G G . G G G G G G G G G G G G G G G G G G G C G G G G G G G G    
984	. . . . . . . . . . . . . . . . . . . . . . . . . . . . . . . . . . . . . . . . . . A A A A . A A A A A A A A A A A A A A A A A A A . A A A A A A A A    
985	. . . . . . . . . . . . . . . . . . . . . . . . . . . . . . . . . . . . . . . . . . C C C C . C C C C C C C C C C C C C C C C C C C . C C C C C C C C    
986	. . . . . . . . . . . . . . . . . . . . . . . . . . . . . . . . . . . . . . . . . . A A A A . A A A A A A A A A A A A A A A A A A A . A A A A A A A A    
987	. . . . . . . . . . . . . . . . . . . . . . . . . . . . . . . . . . . . . . . . . . A A A A . A A A A A A A A A A A A A A A A A A A . A A A A A A A A    
988	. . . . . . . . . . . . . . . . . . . . . . . . . . . . . . . . . . . . . . . . . . C C C C . C C C C C C C C C C C C C C C C C C C . C C C C C C C C    
989	. . . . . . . . . . . . . . . . . . . . . . . . . . . . . . . . . . . . . . . . . . A A A A . A A C A A A A A A A A A A A A A A A A . A A A A A A A A    
990	. . . . . . . . . . . . . . . . . . . . . . . . . . . . . . . . . . . . . . . . . . C C C C . C C . C C C C C C C C C C C C C C C C . C C C C C C C C    
991	. . . . . . . . . . . . . . . . . . . . . . . . . . . . . . . . . . . . . . . . . . A A A A . A A . A A A A A A A A A A A A A A A A . A A A A A A A A    
992	. . . . . . . . . . . . . . . . . . . . . . . . . . . . . . . . . . . . . . . . . . C C C C . C C . C C C C C C C C C C C C C C C C . C C C C C C C C    
993	. . . . . . . . . . . . . . . . . . . . . . . . . . . . . . . . . . . . . . . . . . G G G C . G G . G G G G G G G G G G G G G G G G . G G G G G G G G    
994	. . . . . . . . . . . . . . . . . . . . . . . . . . . . . . . . . . . . . . . . . . A A A . . A A . A A A A A A A A A A A A A A A A . A A A A A A A A    
995	. . . . . . . . . . . . . . . . . . . . . . . . . . . . . . . . . . . . . . . . . . A A A . . A A . A A A A A A A A A A A A A A A A . A A A A A A A A    
996	. . . . . . . . . . . . . . . . . . . . . . . . . . . . . . . . . . . . . . . . . . C C C . . C C . C C C C C C C C C C C C C C C C . C C C C C C C C    
997	. . . . . . . . . . . . . . . . . . . . . . . . . . . . . . . . . . . . . . . . . . C C C . . C C . C C C C G G C C C G G G G G G G . G G G G G G G G  12	 transversion  
998	. . . . . . . . . . . . . . . . . . . . . . . . . . . . . . . . . . . . . . . . . . . . . . . . . . . . . . C C . . . C C C C C C C . C C C C C C C C    
999	. . . . . . . . . . . . . . . . . . . . . . . . . . . . . . . . . . . . . . . . . . . . . . . . . . . . . . C C . . . C C A A C C C . C A A A A A A A  8	 transversion  
1000	. . . . . . . . . . . . . . . . . . . . . . . . . . . . . . . . . . . . . . . . . . . . . . . . . . . . . . . . . . . . . G G . . . . . G G G G G G G    
1001	. . . . . . . . . . . . . . . . . . . . . . . . . . . . . . . . . . . . . . . . . . . . . . . . . . . . . . . . . . . . . A A . . . . . A A A A A A A    
1002	. . . . . . . . . . . . . . . . . . . . . . . . . . . . . . . . . . . . . . . . . . . . . . . . . . . . . . . . . . . . . T T . . . . . T T T T T T T    
1003	. . . . . . . . . . . . . . . . . . . . . . . . . . . . . . . . . . . . . . . . . . . . . . . . . . . . . . . . . . . . . C C . . . . . C C C C C C C    
1004	. . . . . . . . . . . . . . . . . . . . . . . . . . . . . . . . . . . . . . . . . . . . . . . . . . . . . . . . . . . . . C C . . . . . C T T T T T T    
1005	. . . . . . . . . . . . . . . . . . . . . . . . . . . . . . . . . . . . . . . . . . . . . . . . . . . . . . . . . . . . . . . . . . . . . T T T T T T    
1006	. . . . . . . . . . . . . . . . . . . . . . . . . . . . . . . . . . . . . . . . . . . . . . . . . . . . . . . . . . . . . . . . . . . . . T T T T T T    
1007	. . . . . . . . . . . . . . . . . . . . . . . . . . . . . . . . . . . . . . . . . . . . . . . . . . . . . . . . . . . . . . . . . . . . . T T T T T T    
1008	. . . . . . . . . . . . . . . . . . . . . . . . . . . . . . . . . . . . . . . . . . . . . . . . . . . . . . . . . . . . . . . . . . . . . . T T T T T    
1009	. . . . . . . . . . . . . . . . . . . . . . . . . . . . . . . . . . . . . . . . . . . . . . . . . . . . . . . . . . . . . . . . . . . . . . T T T T T    
1010	. . . . . . . . . . . . . . . . . . . . . . . . . . . . . . . . . . . . . . . . . . . . . . . . . . . . . . . . . . . . . . . . . . . . . . A A A A A    
1011	. . . . . . . . . . . . . . . . . . . . . . . . . . . . . . . . . . . . . . . . . . . . . . . . . . . . . . . . . . . . . . . . . . . . . . A A A A A    
1012	. . . . . . . . . . . . . . . . . . . . . . . . . . . . . . . . . . . . . . . . . . . . . . . . . . . . . . . . . . . . . . . . . . . . . . C C C C C    
1013	. . . . . . . . . . . . . . . . . . . . . . . . . . . . . . . . . . . . . . . . . . . . . . . . . . . . . . . . . . . . . . . . . . . . . . G G G G G    
1014	. . . . . . . . . . . . . . . . . . . . . . . . . . . . . . . . . . . . . . . . . . . . . . . . . . . . . . . . . . . . . . . . . . . . . . T T T T T    
1015	. . . . . . . . . . . . . . . . . . . . . . . . . . . . . . . . . . . . . . . . . . . . . . . . . . . . . . . . . . . . . . . . . . . . . . C A A A A    
1016	. . . . . . . . . . . . . . . . . . . . . . . . . . . . . . . . . . . . . . . . . . . . . . . . . . . . . . . . . . . . . . . . . . . . . . . T T T T    
1017	. . . . . . . . . . . . . . . . . . . . . . . . . . . . . . . . . . . . . . . . . . . . . . . . . . . . . . . . . . . . . . . . . . . . . . . T T T T    
1018	. . . . . . . . . . . . . . . . . . . . . . . . . . . . . . . . . . . . . . . . . . . . . . . . . . . . . . . . . . . . . . . . . . . . . . . T T T T    
1019	. . . . . . . . . . . . . . . . . . . . . . . . . . . . . . . . . . . . . . . . . . . . . . . . . . . . . . . . . . . . . . . . . . . . . . . C C C C    
1020	. . . . . . . . . . . . . . . . . . . . . . . . . . . . . . . . . . . . . . . . . . . . . . . . . . . . . . . . . . . . . . . . . . . . . . . C C C C    
1021	. . . . . . . . . . . . . . . . . . . . . . . . . . . . . . . . . . . . . . . . . . . . . . . . . . . . . . . . . . . . . . . . . . . . . . . G G G G    
1022	. . . . . . . . . . . . . . . . . . . . . . . . . . . . . . . . . . . . . . . . . . . . . . . . . . . . . . . . . . . . . . . . . . . . . . . A A A A    
1023	. . . . . . . . . . . . . . . . . . . . . . . . . . . . . . . . . . . . . . . . . . . . . . . . . . . . . . . . . . . . . . . . . . . . . . . T T T T    
1024	. . . . . . . . . . . . . . . . . . . . . . . . . . . . . . . . . . . . . . . . . . . . . . . . . . . . . . . . . . . . . . . . . . . . . . . T T T T    
1025	. . . . . . . . . . . . . . . . . . . . . . . . . . . . . . . . . . . . . . . . . . . . . . . . . . . . . . . . . . . . . . . . . . . . . . . C C C C    
1026	. . . . . . . . . . . . . . . . . . . . . . . . . . . . . . . . . . . . . . . . . . . . . . . . . . . . . . . . . . . . . . . . . . . . . . . C C C C    
1027	. . . . . . . . . . . . . . . . . . . . . . . . . . . . . . . . . . . . . . . . . . . . . . . . . . . . . . . . . . . . . . . . . . . . . . . A A C C    
1028	. . . . . . . . . . . . . . . . . . . . . . . . . . . . . . . . . . . . . . . . . . . . . . . . . . . . . . . . . . . . . . . . . . . . . . . C G . .    
1029	. . . . . . . . . . . . . . . . . . . . . . . . . . . . . . . . . . . . . . . . . . . . . . . . . . . . . . . . . . . . . . . . . . . . . . . C . . .    
1030	. . . . . . . . . . . . . . . . . . . . . . . . . . . . . . . . . . . . . . . . . . . . . . . . . . . . . . . . . . . . . . . . . . . . . . . C . . .    
1031	. . . . . . . . . . . . . . . . . . . . . . . . . . . . . . . . . . . . . . . . . . . . . . . . . . . . . . . . . . . . . . . . . . . . . . . C . . .    
1032	. . . . . . . . . . . . . . . . . . . . . . . . . . . . . . . . . . . . . . . . . . . . . . . . . . . . . . . . . . . . . . . . . . . . . . . C . . .
```
